# Non-destructive quantification of anaerobic gut fungi and methanogens in co-culture reveals increased fungal growth rate and changes in metabolic flux relative to mono-culture

**DOI:** 10.1186/s12934-021-01684-2

**Published:** 2021-10-18

**Authors:** Patrick A. Leggieri, Corey Kerdman-Andrade, Thomas S. Lankiewicz, Megan T. Valentine, Michelle A. O’Malley

**Affiliations:** 1 Department of Chemical Engineering, University of California, Santa Barbara, CA 93106 USA; 2 Department of Ecology, Evolution, and Marine Biology, University of California, Santa Barbara, CA 93106 USA; 3 Joint BioEnergy Institute (JBEI), Emeryville, CA 94608 USA; 4 Department of Mechanical Engineering, University of California, Santa Barbara, CA 93106 USA

**Keywords:** Anaerobic fungi, Methanogens, Co-culture concentrations, Lignocellulose, CAZymes, Hydrogenosome, Synthetic consortia, Co-culture growth rates, Metabolic flux, Non-model microbes

## Abstract

**Background:**

Quantification of individual species in microbial co-cultures and consortia is critical to understanding and designing communities with prescribed functions. However, it is difficult to physically separate species or measure species-specific attributes in most multi-species systems. Anaerobic gut fungi (AGF) (Neocallimastigomycetes) are native to the rumen of large herbivores, where they exist as minority members among a wealth of prokaryotes. AGF have significant biotechnological potential owing to their diverse repertoire of potent lignocellulose-degrading carbohydrate-active enzymes (CAZymes), which indirectly bolsters activity of other rumen microbes through metabolic exchange. While decades of literature suggest that polysaccharide degradation and AGF growth are accelerated in co-culture with prokaryotes, particularly methanogens, methods have not been available to measure concentrations of individual species in co-culture. New methods to disentangle the contributions of AGF and rumen prokaryotes are sorely needed to calculate AGF growth rates and metabolic fluxes to prove this hypothesis and understand its causality for predictable co-culture design.

**Results:**

We present a simple, microplate-based method to measure AGF and methanogen concentrations in co-culture based on fluorescence and absorbance spectroscopies. Using samples of < 2% of the co-culture volume, we demonstrate significant increases in AGF growth rate and xylan and glucose degradation rates in co-culture with methanogens relative to mono-culture. Further, we calculate significant differences in AGF metabolic fluxes in co-culture relative to mono-culture, namely increased flux through the energy-generating hydrogenosome organelle. While calculated fluxes highlight uncertainties in AGF primary metabolism that preclude definitive explanations for this shift, our method will enable steady-state fluxomic experiments to probe AGF metabolism in greater detail.

**Conclusions:**

The method we present to measure AGF and methanogen concentrations enables direct growth measurements and calculation of metabolic fluxes in co-culture. These metrics are critical to develop a quantitative understanding of interwoven rumen metabolism, as well as the impact of co-culture on polysaccharide degradation and metabolite production. The framework presented here can inspire new methods to probe systems beyond AGF and methanogens. Simple modifications to the method will likely extend its utility to co-cultures with more than two organisms or those grown on solid substrates to facilitate the design and deployment of microbial communities for bioproduction and beyond.

**Supplementary Information:**

The online version contains supplementary material available at 10.1186/s12934-021-01684-2.

## Background

Microbial communities continue to attract significant attention from researchers in microbiology, engineering, agriculture, medicine, and beyond owing to their ability to perform seemingly limitless chemical transformations [1]. Physical and metabolic interactions in microbial communities present challenges for quantifying population-specific growth rates, metabolic fluxes, and other characteristic metrics. Developing easy, rapid, and non-invasive methods to characterize consortium membership is critical. However, microbes in natural consortia are difficult to physically separate and can even form biofilms [2], making colorimetric or spectroscopic methods difficult to deploy. Here, we describe the identification of microbe-specific spectroscopic signals that enable quantification of growth rates and fluxes in co-cultures of anaerobic gut fungi (AGF) and methanogenic archaea (methanogens). Ultimately, these metrics enable testing of hypotheses related to their biomass valorization performances in co-culture relative to mono-culture.

AGF native to the rumen of large herbivores have promise for sustainable and economical degradation of lignocellulosic biomass and conversion to value-added products such as pharmaceuticals and commodity chemicals [3], especially if they can be deployed in consortia with other rumen-native microbes. AGF possess nature’s greatest quantity and variety of biomass-degrading carbohydrate-active enzymes (CAZymes) [4], which are readily produced in laboratory culture to degrade a variety of lignocellulose, polysaccharide, oligosaccharide, and monosaccharide substrates for downstream conversion to value-added products [3, 5]. AGF physically associate and exchange metabolites with bacteria and methanogens in the rumen [6–8], leading some to suggest that interactions between AGF and prokaryotes significantly enhance both the rate and extent of biomass degradation relative to isolated AGF [9, 10].

To leverage this effect for industrial bioproduction, researchers have formed “top-down” microbial consortia via laboratory culture of microbes enriched from herbivore fecal samples [11]. While communities with AGF and prokaryotes outperform AGF mono-cultures in biogas production rate [11] and show increased biomass degradation relative to solely prokaryotic communities [12], the mechanisms (gene regulation, flux redirection, etc.) that yield these desired outcomes are difficult to probe [1]. Top-down rumen-derived consortia exhibit interwoven syntrophies that could inform model-based design of simpler, more tractable communities with prescribed functions. Quantification of growth rates and metabolic fluxes for populations in complex consortia is imperative for disentangling cross-feeding relationships but challenging to accomplish.

**Fig. 1:**
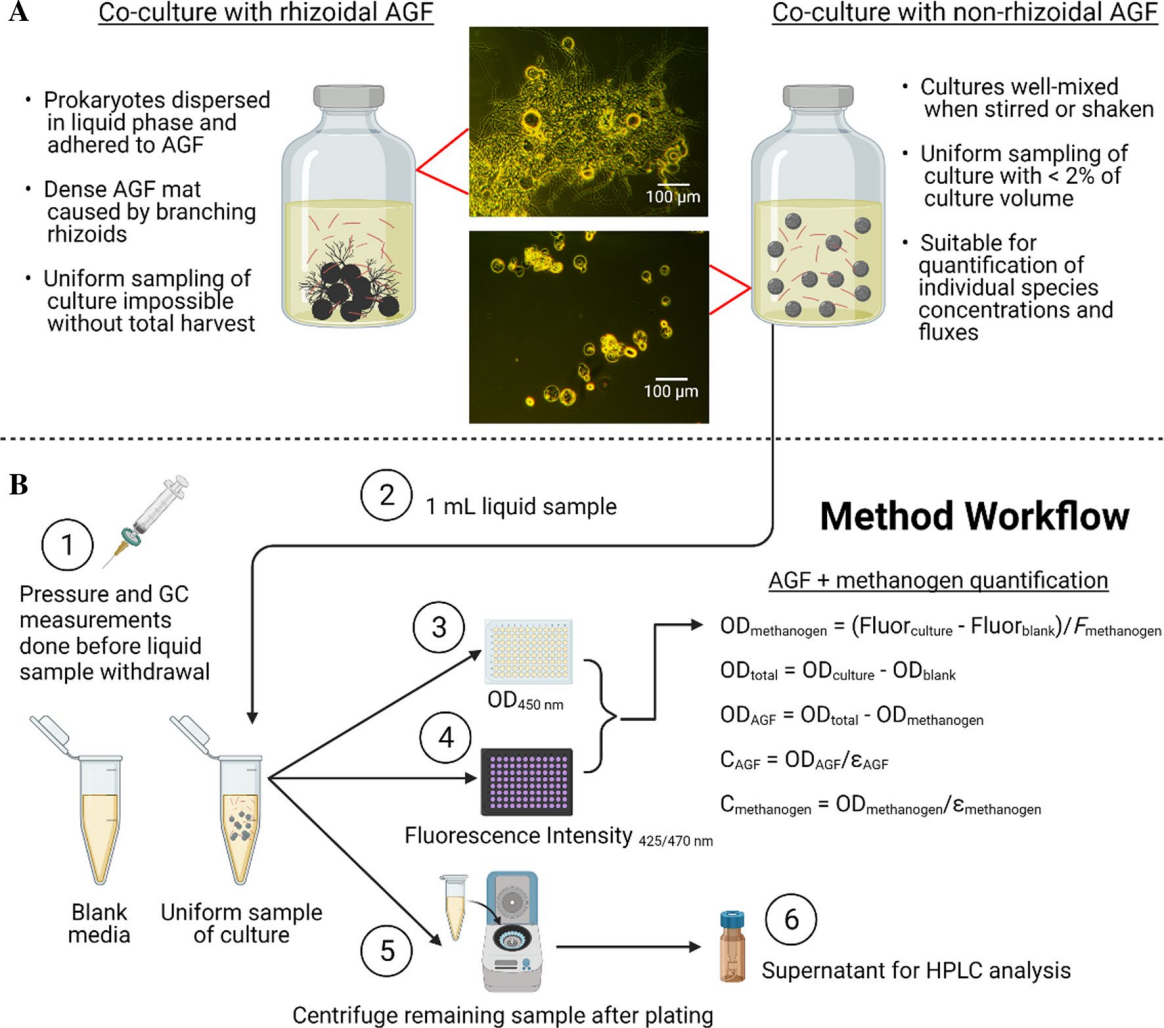
Illustrations of biofilm-like morphology of rhizoidal AGF which cannot be uniformly sampled to track growth in co-culture, and non-rhizoidal AGF such as *C. churrovis*, which form well-mixed co-cultures when shaken or stirred and enable tracking of both species’ concentrations (**A**) via the method outlined in **B**. Created with BioRender

“Bottom-up” assembly of synthetic consortia, in which species are isolated from enrichments and subsequently recombined, offers a way to probe two and three-member interactions in systems that are easier to characterize and model. Insights gained from these more tractable systems help to identify strategies to engineer larger, potentially more robust microbial communities. However, even in two-member co-cultures including AGF, the biofilm-like morphology of AGF caused by their extensive extracellular rhizoidal network (Fig. 1A; Additional file 1) and their physical associations with prokaryotes preclude non-destructive quantification of each species, obscuring how co-culturing with prokaryotes alters the growth rate, per-cell metabolic activity, and CAZyme secretion associated with AGF.

In co-cultures with AGF, methanogens remove putatively inhibitory AGF fermentation products such as formate and hydrogen [13, 14], which might accelerate AGF growth and biomass deconstruction. However, published results are mixed regarding whether these co-cultures show significantly different rates of biomass degradation relative to AGF mono-cultures [10, 11, 15–20], and have been unable to quantify individual species growth rates or cell mass-normalized metabolic fluxes [21]. These metrics directly affect the rate and extent of biomass degradation and the profile of metabolites produced; therefore, they are irreplaceable if anaerobic communities with AGF are to be deployed for bioproduction [22], especially if predictive metabolic models are to be developed for co-cultures with AGF [23]. Quantitative polymerase chain reaction (qPCR)-based methods may be developed to estimate species concentrations in microbial communities [24, 25], and researchers have used them to quantify AGF in mono-culture [26]; however, these methods require thorough optimization, and can be time consuming to run, motivating the development of simpler methods.

In this method, we leverage the non-rhizoidal morphology of the AGF species, *Caecomyces churrovis* [27], to form homogeneous shaken or stirred synthetic co-cultures of AGF with the autofluorescent rumen methanogen, *Methanobrevibacter thaueri* [28], that can be sampled for growth and flux quantification by harvesting as little as 0.5 mL of the culture at each timepoint (Fig. 1A). Despite lacking extracellular rhizoids, *C. churrovis* produces a quantity and variety of biomass degrading enzymes comparable to rhizoidal AGF [27]. *C. churrovis* is metabolically similar to other AGF, as 97 % of its enzyme commission (EC) numbers are shared with at least one other AGF, making it a promising model AGF (Additional file 2).

We utilize non-interfering fluorescence intensity (characteristic of all methanogens) and optical density measurements to resolve the concentrations of both species in co-culture simultaneously, unlocking growth curves and metabolic fluxes for AGF in co-culture with prokaryotes for the first time. To date, measurements of gas accumulation have been the standard for indirectly tracking AGF growth. Optical density measurements offer a simple, more direct measurement of AGF concentration. We test and validate our method by assessing whether co-culturing with *M. thaueri* significantly alters the growth rate or metabolic flux of *C. churrovis* in defined media on both mono- and polysaccharide substrates. Concentration-normalized AGF metabolic flux measurements highlight major discrepancies with widely-accepted models of the AGF hydrogenosome, an energy-generating organelle directly involved in metabolite exchange with methanogens and production of biorefinery precursors including formate and acetate. Further, limitations of the method are discussed, including how it may be extended to quantify individual species populations in microbial communities with more or different species, as well as those without an autofluorescent organism. Ultimately, this method enables per-species measurements that are critical for the design and deployment of biotechnologically-relevant microbial communities.

## Materials and methods

### Culture of anaerobic gut fungi and methanogens

A modified version of anaerobic Medium B (MB) [29] was used for both routine culture and growth experiments of AGF and methanogens (Additional file 3); methanogen mono-cultures received the yeast extract and casitone/peptone supplements, and AGF mono- and co-cultures were grown in the fully defined formulation. The headspace of AGF mono- and co-cultures was 5% H_2_, 35% CO_2_, balance N_2_, and the headspace of methanogen mono-cultures was 80% H_2_, balance CO_2_. All cultures were grown at 39 ºC in 75 mL anaerobic serum bottles with 45 mL of liquid medium. AGF were grown on the soluble carbon sources glucose (anhydrous, Thermo Fisher Scientific, Waltham, MA, USA) or xylan (from corn core, TCI America, Portland, OR, USA) at final concentrations of 5 g/L.

*Caecomyces churrovis* was previously isolated from the feces of a large herbivore [27], and isolated *Methanobrevibacter thaueri* was purchased from DSMZ (DSM 11995). For routine culture, *C. churrovis* was transferred to new media every 2–4 days, and *M. thaueri* was transferred every 4–10 days. In all growth experiments, starter cultures of AGF and methanogens were grown for 48 h and used for inoculation. All inoculums were 10 % v/v. Growth of AGF and methanogens was monitored using the pressure accumulation method described previously [5] and the combined fluorescence intensity and optical density method outlined in Fig. 1. Each condition (co-culture and mono-culture) was grown in biological triplicate, alongside three blank media bottles.

### High performance liquid chromatography (HPLC) analysis of sugars and metabolites

Sulfuric acid (50 mM) was added (10% v/v) to AGF hydrolysate supernatant samples that were then vortexed, held at room temperature for 5 min, then centrifuged for 5 min at 21,000 g. The supernatants were then dispensed into HPLC vials and run on an Agilent 1260 Infinity HPLC (Agilent, Santa Clara, CA, USA) using a Bio-Rad Aminex HPX-87 H column (Part No. 1250140, Bio-Rad, Hercules, CA, USA) with an inline 0.22 μm filter (Part No. 50671551, Agilent) followed by a Micro-Guard Cation H guard column (Part No. 1250129, Bio-Rad, Hercules, CA, USA) before the analytical column. Samples were run with a 5 mM sulfuric acid mobile phase at a flow rate of 0.6 mL/min and a column temperature of 50 ºC. Glucose, xylan, and ethanol were detected using a refractive index detector; succinate, lactate, formate, fumarate, and acetate were detected using a variable wavelength detector set to 210 nm. Standards were created for all sugars and metabolites in deionized water at 1%, 0.1%, and 0.01% w/v concentrations and the above protocol was followed to run each standard. Standard curve R^2^ values ranged from 0.9996 to 1.000.

Metabolic flux measurements for each metabolite were calculated based on measurements one and two days after inoculation as follows: the difference in amount (mmol) of that metabolite in each culture divided by the average calculated AGF mass (gram dry weight, GDW) present in the culture bottle during that time, divided by the elapsed time between the two measurements. Flux units reported here are mmol GDW^−1^ h^−1^.

### Gas chromatography (GC) analysis of hydrogen and methane

To analyze the headspace composition of each culture at each measurement timepoint, 100 µL of headspace gas was collected and subsequently purged three times in a 100 µL air-tight syringe and needle. Then, 20 µL of headspace gas was collected and injected directly into a Thermo Fisher Scientific TRACE 1300 gas chromatograph (Thermo Fisher Scientific) with a TracePLOT™ TG-BOND Msieve 5 A (Part No. 26003-6100, Thermo Fisher Scientific) and an Instant Connect Pulsed Discharge Detector (PDD) (Part No. 19070014, Thermo Fisher Scientific). The oven temperature for each run was 30 ºC and the PDD temperature was 150 ºC. High-purity helium (Part No. HE 5.0UH-55, Praxair, Danbury, CT, USA) was further purified with a heated helium purifier (Part No. HP2, VICI) and used as the carrier gas with a flow rate of 0.5 mL/min. The same flushing and analysis procedures were followed for methane and hydrogen standards including 500 ppm H_2_, 2% H_2_, 5 % H_2_, 20% H_2_, 0.5 % CH_4_, 1% CH_4_, 5% CH_4_, 10% CH_4_, and 20 % CH_4_ with balance helium (Douglas Fluid & Integration Technology, Prosperity, SC), which were run at each measurement timepoint to account for the PDD baseline that varied slightly each day. Standard curve R^2^ values ranged from 0.7370 to 0.9979.

### Quantification of anaerobic fungi and methanogens via plate reader and lyophilizer

AGF and methanogens were quantified with optical density at 450 nm and fluorescence intensity (excitation/emission: 425 nm/ 470 nm, bandwidth 10 nm) using a Tecan M1000 Infinite Microplate Reader (Tecan, Männedorf, Switzerland). Fluorescence intensity measurements were obtained with a manual gain setting of 77 for each sample and blank and were normalized by the fluorescence intensity of aliquots of Pacific Blue dye (100 µg/L) (succinimidyl ester, Thermo Fisher Scientific, Canoga Park, CA, USA). UV-transparent 96 well plates (Part No. 3635, Corning, Corning, NY, USA) were used for optical density measurements, and black MicroFluor2 96 well plates were used for fluorescence intensity measurements (Part No. 437111, Thermo Fisher Scientific).

The dry cell weights of AGF and methanogens in culture vessels at the end of growth were determined by harvesting and centrifuging the cultures (10,000 g for 20 min) in tared centrifuge tubes, washing the cell pellets with deionized water and centrifuging again, lyophilizing for 48 h in a FreeZone 4.5 L Benchtop Freeze Dry System (Part No. 77500200, Labconco Corp., Kansas City, MO, USA), and weighing the dried samples in the centrifuge tubes.

### Microscopy

Micrographs of AGF and methanogens were captured with a Leica SP8 resonant scanning confocal microscope (Leica Microsystems, Wetzlar, Germany) with photomultiplier tube (PMT) and HyD detectors and 405 nm, argon, and white light lasers. AGF were imaged using the white light laser and transmitted light PMT to collect brightfield images, and methanogens were imaged using the 405 nm excitation laser with HyD detector set to detect emission between 460 and 480 nm. Images were collected and analyzed using the LAS X Life Science Microscope Software Platform (Leica Microsystems). Samples were imaged without fixation using a slide and coverslip. A 20× water objective (numerical aperture = 0.75) was used to collect all images presented here. Lateral magnification was 284 nm/pixel.

### Statistical analysis

All statistical analyses were conducted using the Prism 9.1.2 software (GraphPad, San Diego, CA, USA). Prism 9.1.2 was used to (i) interpolate the concentrations of metabolites detected via HPLC and GC using standard curves, (ii) determine significant differences in growth rates and metabolite fluxes between growth conditions via *t*-tests, (iii) compare calculated and measured co-culture concentrations via *t*-tests, and (iv) to determine the significance of the slopes and intercepts of linear regressions. In all statistical tests, α = 0.05 was used.

## Results and discussion

### Development of a non-destructive co-culture species quantification method

All AGF with PacBio-sequenced genomes to date, except for *C. churrovis*, grow in dense, biofilm-like mats that cannot be uniformly sampled, precluding direct measurement of cell concentrations without harvesting and weighing the entire culture [29, 30]. Therefore, growth of AGF in mono-culture is typically tracked via pressure accumulation in sealed culture vessels, as AGF produce hydrogen and likely carbon dioxide as they grow [14, 30]. However, uncertainties in the regulation of and relative flux through gas-generating pathways coupled with the pH-dependent, gas-evolving bicarbonate buffer present in most AGF media [30, 31] make pressure accumulation an indirect measure of AGF growth. Using pressure as a proxy for AGF growth precludes analysis of per-cell hydrogen and carbon dioxide production and quantification of AGF concentration in continuous or even semi-batch cultivation systems, both of which are critical for the eventual deployment of AGF for industrial biotechnology. Further, because methanogens utilize hydrogen and carbon dioxide gasses as well as formate produced by AGF to synthesize methane, it is not possible to account for the total moles of gas produced by AGF in co-culture, preventing pressure-based tracking of the growth of either species in co-culture altogether.

Quantification of two species simultaneously in co-culture requires two independent signals that scale linearly with the concentration of either species or the concentration of the total co-culture. If a signal scales with the concentration of one species, but not the other, then the presence of the other species must not interfere with the signal from the first. Further, the co-culture must be well-mixed enough to enable uniform sampling, and the signals must be measurable with a small enough sample of the culture that growth is not disturbed. In the well-mixed AGF-methanogen co-cultures studied here, we use fluorescence intensity to quantify the methanogen and optical density to quantify the total mass concentration of the co-culture. We use the linear relationship between fluorescence intensity and absorbance during exponential phase in the methanogen to calculate the contribution of the methanogen to the total optical density signal, enabling calculation of the population-specific optical density and therefore concentration of the AGF. The equations and propagation of uncertainty associated with the method are given in Fig. 1B and Additional file 4, respectively.

To track growth and metabolite production in mono- and co-cultures, the workflow outlined in Fig. 1B was conducted at each timepoint. First, the accumulated pressure was measured for each culture vessel and blank media vessel. Next, the headspace gas of each culture vessel was sampled and analyzed via GC. Then, 1 mL of each well-mixed culture vessel and blank was sampled with a needle and syringe and transferred to a microcentrifuge tube. Each tube was vortexed briefly, then pipetted into two separate wells each on clear microplates for measurements of optical density and black microplates for measurements of fluorescence. The remaining volume in each microcentrifuge tube was centrifuged, and the supernatant removed and stored at − 80 º C for HPLC analysis. Finally, the culture and blank vessels were vented to 1 psig.

#### Normalized fluorescence intensity quantifies absolute methanogen concentration in co-culture

Methanogens are quantifiable with autofluorescence intensity due to the fluorescent coenzyme F_420_, which is present in all methanogens and is involved in all three major routes of anaerobic methanogenesis (hydrogenotrophic, acetoclastic, and methylotrophic) [32]. The fluorescence spectrum of coenzyme F_420_ is well-characterized, with expected peak excitation and emission wavelengths near 420 nm and 470 nm, respectively [33]. The intracellular F_420_ content has been shown to be constant in methanogens during exponential-phase growth [34, 35], supporting the use of fluorescence intensity as a direct methanogen concentration measurement. Because fluorescence intensity units are arbitrary and values are subject to vary with nuisance variables such as the lamp power in the microplate reader, ambient temperature, etc., we normalize the observed fluorescence intensity of all samples in a run by the observed fluorescence intensity of a freshly thawed aliquot of Pacific Blue dye. Pacific Blue dye has a similar fluorescence spectrum to methanogens (excitation/emission max: 410 nm/ 455 nm) and is subject to the same nuisance variables as the samples. Therefore, the normalized fluorescence intensity of a methanogen culture (F_culture_/F_dye_) may be used as an absolute measurement of methanogen concentration, as long as all aliquots of the Pacific Blue dye are of identical concentration. We observed no change in the fluorescence intensity of Pacific Blue dye dissolved in dimethyl sulfoxide over 15 months of storage at − 20 º C (Additional file 5).

**Fig. 2:**
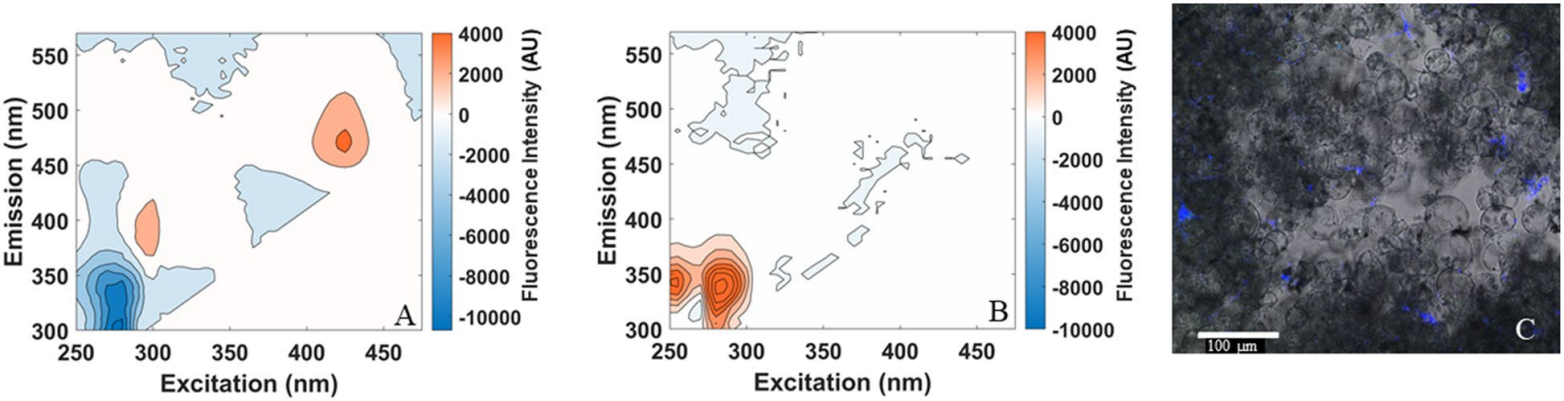
Fluorescence spectra of *M. thaueri* (**A**) and *C. churrovis* (**B**) show 425nm/470nm excitation/emission peak characteristic of all methanogens in *M. thaueri*, and no fluorescence in *C. churrovis* in this channel. Fluorescence intensity is reported as the intensity of the mature mono-culture minus the intensity from an aliquot of blank media. Fluorescence in this channel was observed in a confocal laser scanning fluorescence micrograph (**C**) in *M. thaueri* (blue), but not in *C. churrovis*, which is visible only in the overlaid brightfield micrograph

As seen in Fig. 2A, we observed the expected fluorescence spectrum for *M. thaueri*, with peak excitation and emission wavelengths at 425 and 470 nm, respectively; we observed negligible fluorescence in *C. churrovis* in this channel (Fig. 2B). The micrograph shown in Fig. 2C confirms that *M. thuaeri* is visible in co-culture with *C. churrovis* using a 425/470 nm fluorescent filter, and that *C. churrovis* shows no fluorescence in this channel and is visible only in the brightfield overlay.

**Fig. 3:**
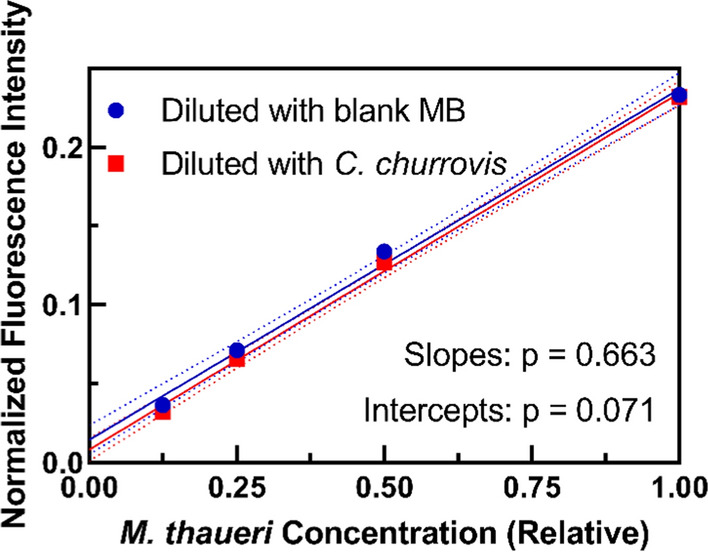
*M. thaueri* (combined pellet and supernatant) normalized fluorescence intensity scales linearly with cell concentration when diluted with blank Medium B, and with a mature *C. churrovis* culture, indicating that *C. churrovis* does not interfere with the fluorescent signal from *M. thaueri*. Dotted lines represent the 95 % confidence interval of each regression. The p-values represent a test for significant differences in the values of the slopes and intercepts of the two regressions

As shown in Fig. 3, the normalized fluorescence intensity of *M. thaueri* mono-cultures scaled linearly with cell concentration when cells were diluted with blank Medium B, and the slope and intercept of this regression was not significantly different when *M. thaueri* was diluted with concentrated *C. churrovis* instead of blank medium (slopes p = 0.663, intercepts p = 0.071). This further verifies that there is no measurable fluorescence of *C. churrovis* in the 425/470 nm channel and demonstrates that the presence of *C. churrovis* does not interfere with the fluorescence signal of *M. thaueri*. These results therefore establish that normalized fluorescence intensity may be used to quantify the absolute concentration of methanogens in co-culture with *C. churrovis* without physically separating the cell populations.

#### Optical density quantifies the concentration of AGF and methanogens in co-culture

Optical density, often at 600 nm, is a well-established measurement of cell concentration for model microbes [36]. However, the dense biofilm-like rhizoidal morphology characteristic of almost all AGF isolated and routinely cultured to date precludes uniform sampling of the culture for quantification via optical density. *C. churrovis* lacks this rhizoidal phenotype and can therefore be uniformly sampled for quantification *via* optical density when grown with constant stirring or when shaken prior to sampling (see Additional file 1 for a visual comparison of rhizoidal AGF and *C. churrovis* morphologies).

**Fig. 4:**
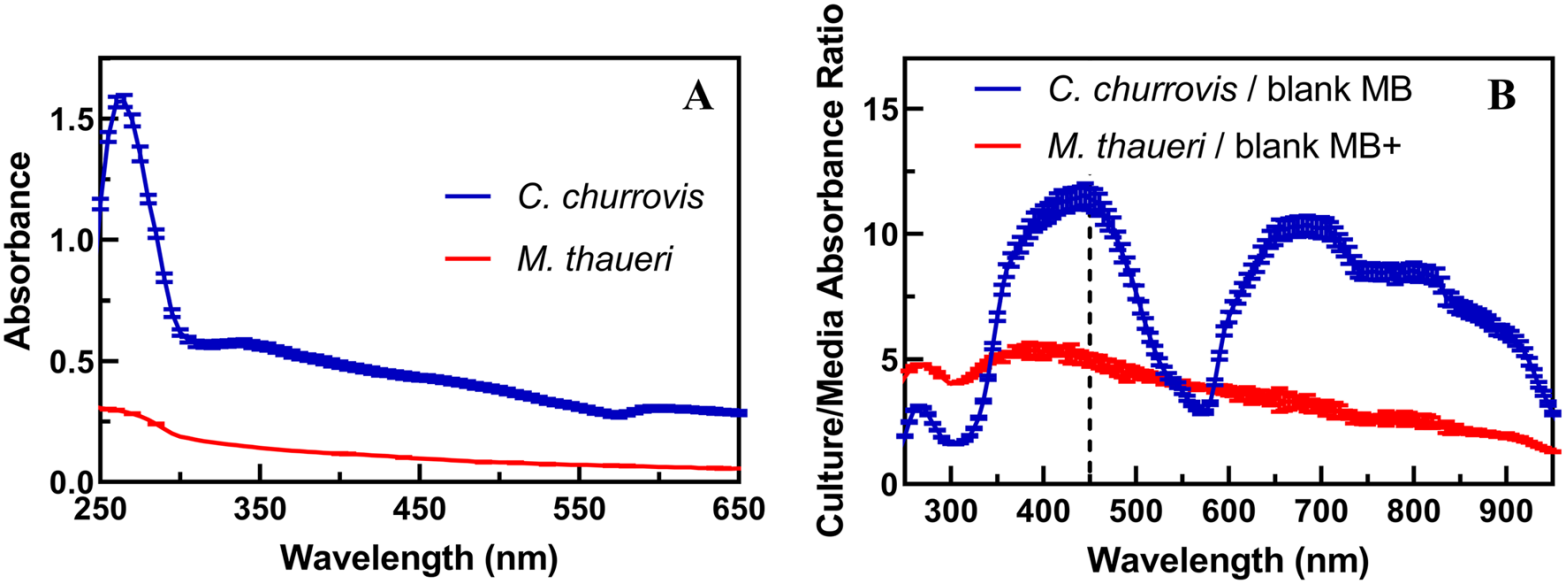
Absorbance spectra of late-exponential phase mono-cultures of *C. churrovis* and *M. thaueri* (with media blanks subtracted) (**A**) show peak values in UV range, but highest culture/media absorbance ratios at or near 450 nm (**B**), highlighted with the dashed line. MB+ indicates the Medium B formulation with yeast extract and casitone added

As seen in Fig. 4A, the peak absorbance values for *C. churrovis* and *M. thaueri* are both near 260 nm. However, the absorbance of blank Medium B is also large in this ultraviolet (UV) region, and the ratio of cell absorbance to media absorbance is at a minimum here (Fig. 4B). Further, variable oxidation states of cofactors and other intracellular metabolites yield appreciable variation in per-cell UV-range absorbances from day to day and batch to batch, making them an unreliable measure of absolute cell concentration. Although the magnitude of absorbance at 450 nm is less than that at 260 nm for both *C. churrovis* and *M. thaueri*, A_450 nm_ scales linearly with cell concentration for both species and offers the largest signal to background media ratio (Fig. 4B**)**, therefore 450 nm was used to determine the total cell concentration in co-cultures.

**Fig. 5:**
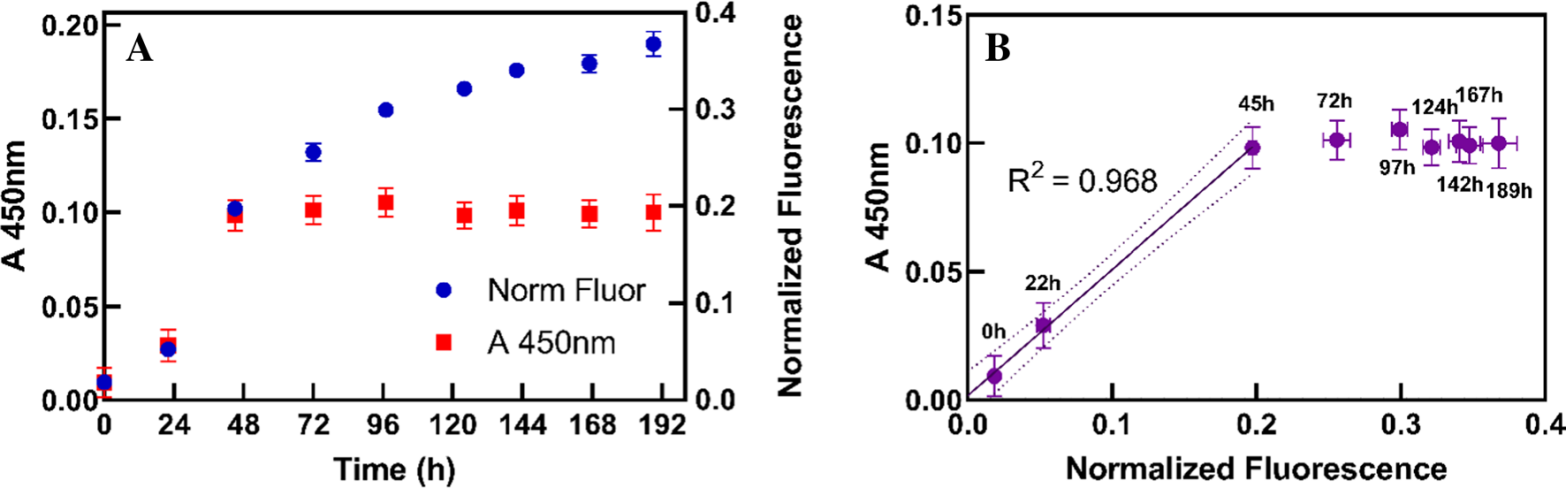
Normalized fluorescence and absorbance at 450 nm scale linearly during exponential phase growth of *M. thaueri* (**A**), in agreement with previously observed constant intracellular coenzyme F_420_ content in methanogens. The high R^2^ value of the regression of absorbance vs. fluorescence during exponential phase (up to 45 h) demonstrates the linearity of the normalized fluorescence:absorbance ratio (**B**). Dotted lines in **B** represent the 95 % confidence interval of the linear regression of absorbance vs. fluorescence during exponential phase

To calculate the optical density of *C. churrovis* in co-culture, we require an estimate of the optical density of *M. thaueri*, which is subtracted from the observed total co-culture optical density (A_450 nm_ of the co-culture) to give the optical density of *C. churrovis* (mathematical steps outlined in Fig. 1B). To estimate the optical density of *M. thaueri* using the normalized fluorescence intensity of the culture, the ratio of normalized fluorescence to absorbance at 450 nm must be constant for *M. thaueri* in mono-culture. As shown in Fig. 5B, the relationship between absorbance and fluorescence intensity is linear (R^2^ = 0.968) during the exponential phase of growth (0–45 h in this case). Therefore, we assume the ratio of normalized fluorescence and absorbance is equal to the average value of the ratio during this period (1.93) for *M. thaueri* in co-culture. As shown in Fig. 5A, normalized fluorescence provides an accurate estimate of the optical density of *M. thaueri* in mono-culture up to the point where the methanogen reaches stationary phase (when absorbance stops increasing, after 45 h in this case). Beyond this point, the fluorescence of the culture continues to increase while the absorbance remains constant, yielding the nonlinear relationship between absorbance and fluorescence after 45 h shown in Fig. 5B.

This divergent relationship between fluorescence and absorbance in stationary phase may be attributable to increased secretion of coenzyme F_420_ by the methanogens in stationary phase relative to exponential phase, and a greater fluorescence intensity of secreted F_420_ than intracellular F_420_. Some previous descriptions of fluorescence-based methanogen quantification recommend removing any culture supernatant and lysing the methanogens to measure only intracellular (and not extracellular) F_420_ [37]. This approach yielded only minor increases in fluorescence intensity compared to the unlysed methanogen pellets (Additional file 6A). Further, unlike the combined pellet and supernatant samples shown in Fig. 3, methanogen pellet fluorescence did not scale linearly with methanogen concentration when diluted with concentrated *C. churrovis* (Additional file 6B). Therefore, extracellular fluorescence was included in quantification of the methanogen in all mono-cultures and co-cultures.

Most batch, semi-batch, and continuous co-cultures prioritize exponential-phase growth; therefore, the divergent ratio of fluorescence to absorbance in methanogens in stationary phase poses minimal practical drawbacks. To accurately calculate stationary phase methanogen concentrations in co-cultures, we assume both species are at stationary phase when the total absorbance of the co-culture stops increasing with time, and assume that the absorbance (and concentration) of the methanogen remains constant at its initial stationary phase value even though fluorescence continues to increase. The ratio of normalized fluorescence to absorbance at 450 nm in *M. thaueri* is based on experimental values, therefore, we treat it as a random variate (1.93 ± 0.13) in all calculations to increase the sensitivity of statistical conclusions drawn based on the calculated concentrations of both species. As the observed fluorescence of a co-culture increases, so too does the uncertainty in the absolute methanogen concentration, and therefore in the AGF concentration as well (Additional file 4).

Table 1 shows the average ratios of optical density at 450 nm to cell concentration (determined *via* measurement of the culture dry weight after lyophilization) for *C. churrovis* and *M. thaueri* in mono-culture from six separate cultures of each species spread across two different batches with different inoculums. The coefficients of variation for both species are below 15 %, and the predicted total mass concentrations for co-cultures based on these correlations are not significantly different from the observed values (Table 2), supporting the accuracy of the method. Correlations are calculated using cell weight rather than cell number because the lytic lifecycle of AGF in which many zoospores develop inside a sporangium complicates the definition and detection of a single fungal “cell” via hemocytometry. While the concentrations of both species are calculable using only the fluorescence intensity and absorbance measurements outlined above, weighing the lyophilized co-cultures is a third, independent metric that may be used to validate the calculated concentrations.

**Table 1:** Individual species absorbance/concentration correlations for six replicate mono-cultures from two different inoculums for each species

Organism	Cell concentration (mg/mL)	Absorbance (450 nm)	Abs/Conc (mL/mg)
*C. churrovis*	0.449 ± 0.043	0.235 ± 0.027	0.523 ± 0.078
*M. thaueri*	0.102 ± 0.015	0.100 ± 0.002	0.997 ± 0.148

Correlations for both species show % coefficients of variation of less than 15%

**Table 2:** Total concentrations of co-cultures grown on xylan and glucose measured via lyophilization (left) and calculated using the individual species absorbance measurements (Fig. 6; Additional file 6) and the absorbance/concentration correlations in Table 1 (right)

Substrate	Measured co-culture concentration (mg/mL)	Calculated co-culture concentration (mg/mL)	p
Xylan	0.472 ± 0.006	0.442 ± 0.028	0.2005
Glucose	0.504 ± 0.016	0.505 ± 0.042	0.9721

On both substrates, the calculated concentration is not significantly different from the measured concentration

#### Potential expansions of the method to other co-culture systems

The method described here may be extended to any co-cultures which can be grown in well-mixed systems and possess two linearly independent signals such as optical density at a given wavelength, fluorescence intensity in a particular excitation/emission channel, fluorescence lifetime, fluorescence polarization, or any other signal that reproducibly scales linearly with the concentration of one species or the total concentration of the co-culture. We leverage methanogen autofluorescence as one of the two signals here; for genetically tractable organisms, fluorescence may be introduced via genetic engineering. However, constant expression of the fluorescent protein over the course of growth would be required, which is particularly difficult in anaerobic systems [38].

In a simpler case, individual species concentrations may be resolved in a co-culture using absorbance signals at two different wavelengths, provided that the ratio of per-cell absorbance between those two wavelengths is different in the two organisms and constant over the course of growth in both organisms. The absorbance profiles of microbes depend on many factors including their size and intracellular composition; therefore it is likely that two linearly independent wavelengths exist for most co-culture pairs, even for prokaryote-prokaryote systems. For tri-cultures, a third linearly independent absorbance wavelength must exist.

For systems grown on solid substrates such as lignocellulosic biomass, the method we present here may still be applied if the culture (with substrate) can be uniformly sampled, and the microorganisms can be subsequently removed from the substrate entirely, potentially with a detergent-based procedure similar to the one described in [39]. Such systems will likely require more samples, and thus larger cultures, to capture the heterogeneity of the multiphase culture, as well as thorough controlling of the background autofluorescence of the substrate and any detergents.

### Co-culturing ***C. churrovis*** with ***M. thaueri*** significantly increases AGF growth rate and xylan and glucose deconstruction rates relative to AGF mono-cultures

We used the method outlined above to determine whether co-culturing with a methanogen increases the growth rate, polysaccharide and monosaccharide deconstruction rates, and mass-normalized flux of key metabolites in *C. churrovis* relative to mono-culture. We use xylan and glucose as the substrates in separate experiments, as they are soluble in Medium B and quantifiable via HPLC, and therefore allow uniform sampling of the culture for quantification of both species and the chemical composition of the supernatant.

**Fig. 6:**
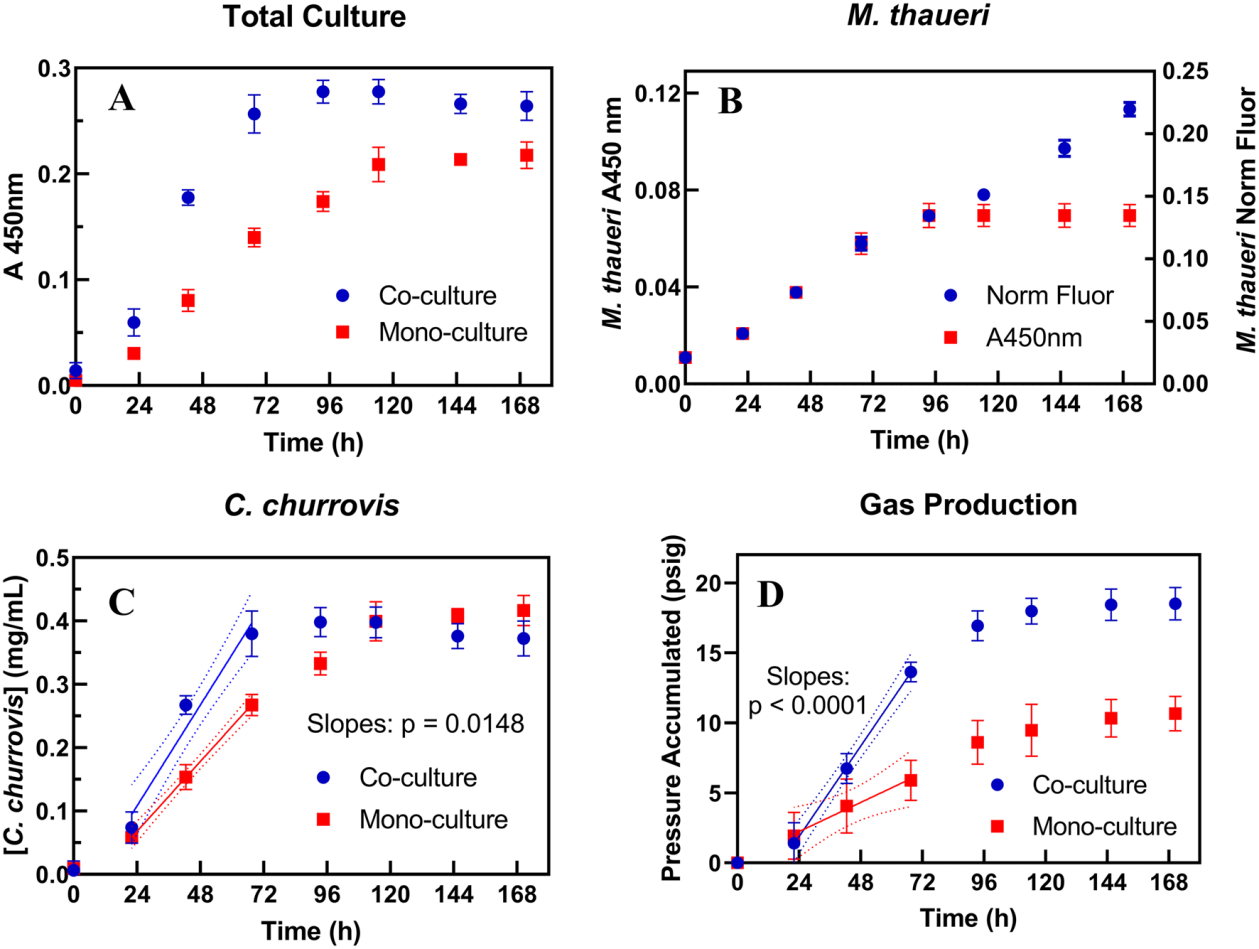
Total culture absorbance (**A**), *M. thaueri* fluorescence + absorbance (**B**), *C. churrovis* concentration (**C**) and accumulated pressure (**D**) curves show that both the growth rate of *C. churrovis* and the rate of gas production are significantly increased in co-cultures with *M. thaueri* grown on xylan, relative to mono-cultures. Panel B shows the divergence of *M. thaueri* fluorescence relative to absorbance in stationary phase also observed in mono-culture; the absorbance of the methanogen was assumed to remain constant after the absorbance of the co-culture stopped increasing (96 h and after). Dotted lines represent the 95% confidence interval of each regression. The p-values in panels **C** and **D** represents a test for significant difference in the values of the slopes of the two regressions

The growth curves of *C. churrovis* and *M. thaueri* as well as the pressure accumulation and total co-culture optical density curves during growth on xylan are shown in Fig. 6. Figure 6A shows that, at all timepoints, the optical density of the co-culture was greater than that of the mono-culture, as expected. Using the measured relationship between fluorescence intensity and optical density for *M. thaueri*, we can estimate how much of the total optical density of the co-culture is attributable to *M. thaueri* (Fig. 6B), and therefore determine the concentration of *C. churrovis* at each timepoint (Fig. 6C). Note that the fluorescence of the co-culture increases throughout stationary-phase growth (after 96 h in this case), but the absorbance of the methanogen is assumed constant, as discussed previously. As seen in Fig. 6C, the slopes of the *C. churrovis* concentration vs. time regressions during the period of approximately constant growth rate are significantly different between co-culture and mono-culture (p = 0.0148), indicating that co-culturing with *M. thaueri* does increase the growth rate of *C. churrovis* on xylan. These data represent the first evidence of a significant difference in growth rates of AGF in synthetic co-culture vs. mono-culture using direct AGF concentration-based measurements. Like previous studies [9], we observe significantly enhanced rates of gas accumulation in co-culture vs. mono-culture (Fig. 6D, p < 0.0001); however, this alone does not demonstrate faster growth of the fungus, even though pressure accumulation curves typically correlate with AGF concentration in mono-culture [29].

**Fig. 7:**
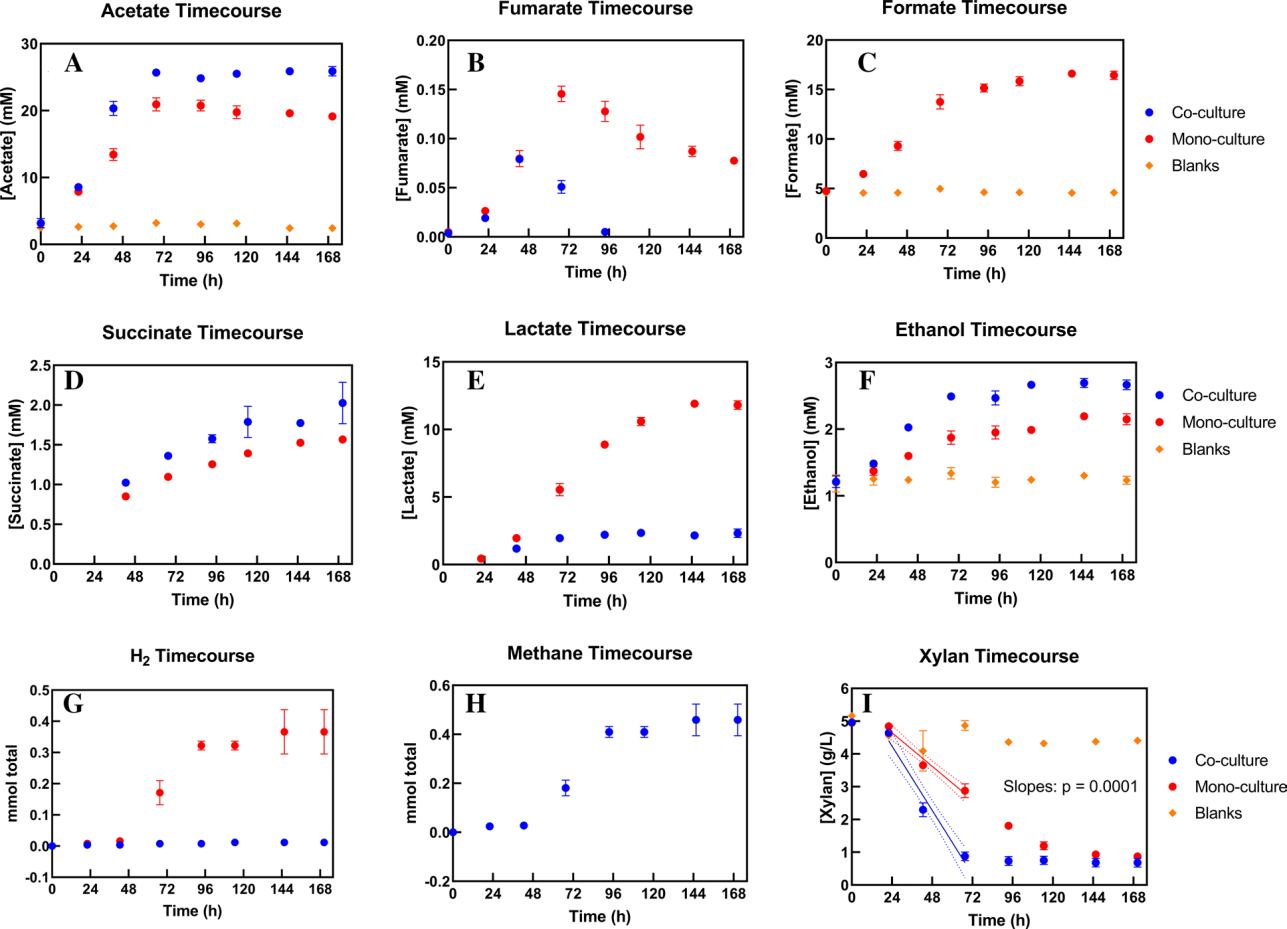
Metabolite profiles for mono- and co-cultures grown on xylan reveal significantly faster xylan degradation in co-culture (panel **I**), negligible hydrogen and formate accumulation in co-culture (panels **C** and **G**), greater lactate production in mono-culture (panel **E**), greater acetate, succinate, and ethanol production in co-culture (panels **A**, **D**, and **F**, respectively), and less accumulation of fumarate in co-culture (panel **B**). Metabolite concentrations at 24 and 43 h shown here were used to calculate fluxes of each metabolite. Dotted lines represent the 95% confidence interval of each regression. The p-value in panel **I** represents a test for significant difference in the values of the slopes of the two regressions

As seen in Fig. 7I, the rate of xylan degradation by AGF was significantly greater in co-culture than mono-culture (p = 0.0001). To the best of our knowledge, this is the first evidence of a significantly greater polysaccharide degradation rate in a synthetic AGF-methanogen co-culture compared to an AGF mono-culture (see the supplement of [11] for a statistical analysis of previous studies related to this conclusion). These data support the previous finding that biomass-degrading CAZymes, including xylanases, were upregulated in co-cultures of AGF and methanogens relative to AGF mono-cultures [40].

Previously, it was shown that neither the rate of sugar release from cellulosic filter paper [11] nor the rate of xylose utilization [21] by AGF were significantly increased by co-culturing with a methanogen. Interestingly, we observed a similar result in an AGF-methanogen co-culture grown on glucose and inoculated with a seven-day-old methanogen culture that was in stationary phase (Additional file 7). Methanogen growth in co-culture was confirmed by increasing fluorescence and production of methane, but neither the growth rate of *C. churrovis* nor the rate of glucose utilization differed from mono-cultures (p = 0.5509, p = 0.1067, respectively). However, as seen in Additional files 8 and 9, when the glucose experiment was repeated with a 2 day-old methanogen inoculum, the growth rate of *C. churrovis* was significantly greater in co-culture than mono-culture (p = 0.0107), the rate of glucose degradation was significantly greater in co-culture (p < 0.0001), and gas productivity was greater in co-culture (p = 0.0025). Some dependence of AGF growth rate on the growth phase of the methanogen inoculum may partially explain the variable results of AGF-methanogen co-cultures in literature.

#### AGF-methanogen co-cultures grown on xylan and on glucose show significantly different mass-normalized metabolic fluxes compared to mono-cultures

**Fig. 8:**
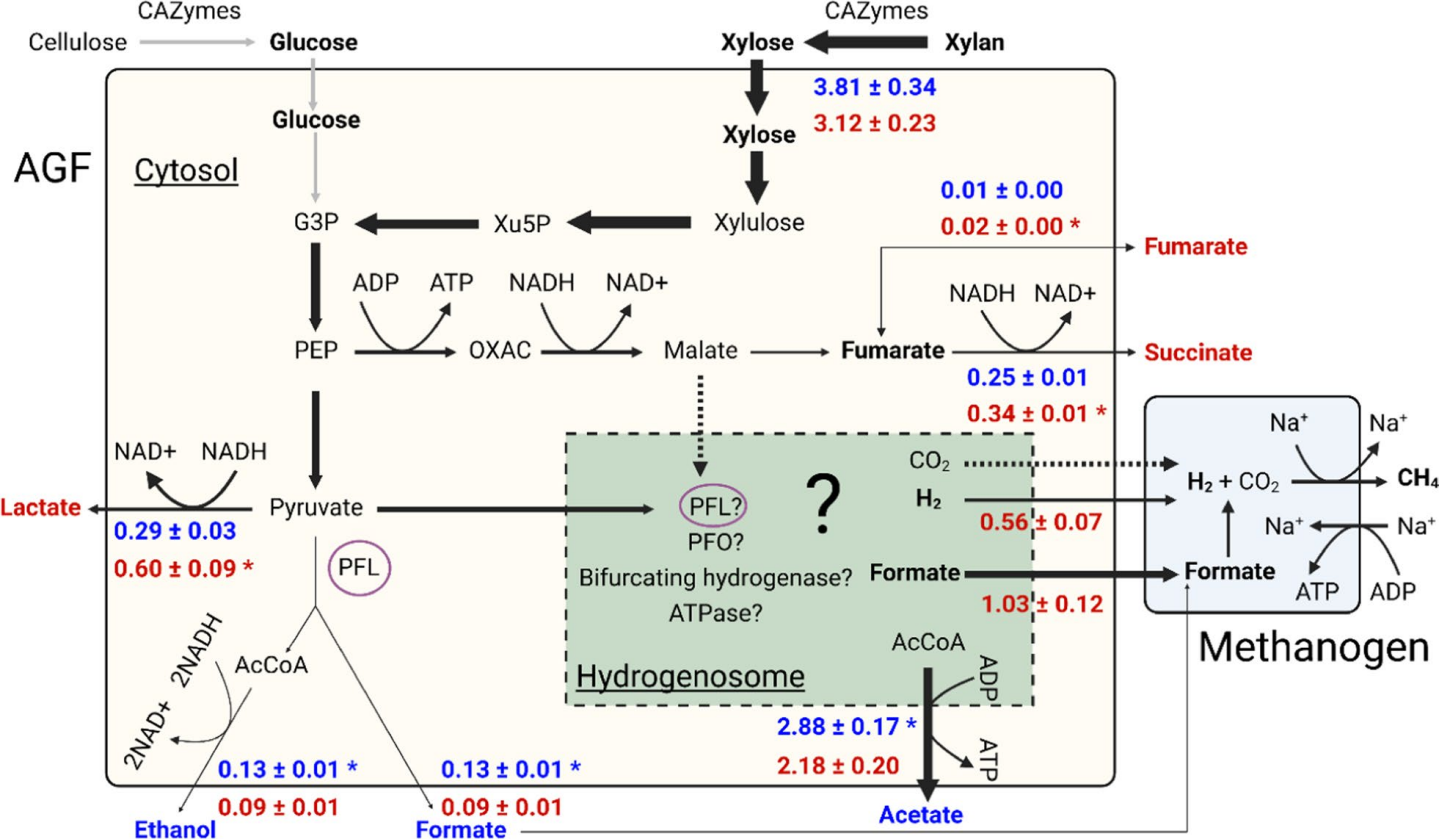
AGF mass-normalized fluxes reveal significant upregulation of acetate (via the hydrogenosome) and ethanol (via cytosolic PFL) fluxes, and significant downregulation of lactate and succinate flux in co-cultures. Formate and hydrogen are consumed by *M. thaueri* and therefore do not accumulate in co-cultures. While xylan is consumed more quickly in co-culture, the flux of xylan into *C. churrovis* is equal in mono- and co-cultures. Fluxes for succinate in mono- and co-culture and lactate in co-culture assume metabolite concentrations of 0 mM at 24 h, as observed values were below the detection limit. Bolded metabolites are detectable via our HPLC method. Metabolites in blue or red (also starred) showed significantly greater flux in co-culture or mono-culture, respectively. Arrow thickness correlates qualitatively with mono-culture flux values. *Xu5P* xylulose-5-phosphate, *G3P* glyceraldehyde-3-phosphate, *PEP* phosphoenolpyruvic acid, *OXAC* oxaloacetic acid, *ADP* adenosine diphosphate, *ATP* adenosine triphosphate, *NAD+* nicotinamide adenine dinucleotide (oxidized), *NADH* nicotinamide adenine dinucleotide (reduced), *AcCoA* acetyl coenzyme A, *PFL* pyruvate formate lyase, *PFO* pyruvate:ferredoxin oxidoreductase. Created with BioRender

The metabolite concentrations in Fig. 7 combined with the *C. churrovis* concentrations in Fig. 6C enable calculation of the flux of each metabolite in mono- and co-cultures grown on xylan. Because fluxes are typically most accurately analyzed in the context of predictive metabolic models during steady-state growth [41], we present fluxes at only one timepoint, 43 h, the middle of the period of approximately constant growth rate. As seen in Fig. 8, significant differences exist between mono- and co-culture for the fluxes of all metabolites measured except for formate (which was utilized by *M. thaueri*^1^, precluding accurate calculation of formate flux in co-culture) and xylan. The lack of difference in xylan flux between mono- and co-culture implies similar growth yields (gDW/mol_xylan_) and per-cell xylan deconstruction activities between the two conditions. Internal fluxes are often compared between conditions by normalizing by the influx of carbon substrate for each condition [42]; in this case, although we are concerned primarily with external fluxes, we may directly compare the absolute flux values of each metabolite in mono- and co-culture because the xylan influxes are similar.

Although the pathways within the AGF hydrogenosome and their relative utilization remain uncertain [43], the fluxes presented in Fig. 8 support the conclusion by Li et al. that co-culturing with methanogens causes AGF to direct more flux through the hydrogenosome [21]. While the hydrogenosome model used in that work, proposed previously by Boxma et al. [44], relies on an energetically unfavorable route of hydrogen production (reduction of protons to hydrogen coupled to regeneration of NAD(P)+ from NAD(P)H), the yield of one mole of acetate per mole of malate or pyruvate that enters the hydrogenosome in that model was supported by a recent genomic and transcriptomic characterization of the AGF hydrogenosome [43]. Because acetate is putatively only produced in AGF in the hydrogenosome, and not in the cytosol *via* acetaldehyde dehydrogenase which would reduce additional NAD+ instead of regenerating it from NADH, we estimate acetate flux as a proxy for hydrogenosome flux (note the uncertainty of hydrogenosomal pathways in Fig. 8). See [43] for a description of all observed hydrogenosomal transcripts in *Neocallimastix lanati*, an AGF that is metabolically similar to *C. churrovis* (84 % of EC numbers shared between both species, Additional file 2). While significantly more work is needed to characterize the AGF hydrogenosome, the significantly greater acetate flux in co-culture than mono-culture (p = 0.0320) implies increased flux through the hydrogenosome in co-culture than in mono-culture.

In support of the increased hydrogenosome vs. cytosol flux in co-culture, as shown in Fig. 8, the fluxes of lactate and succinate, metabolites produced to regenerate oxidized NAD+ from NADH in the cytosol, were significantly lower in co-culture than mono-culture (p = 0.0177, 0.0012, respectively). The external flux of fumarate, an intermediate in cytosolic succinate production, was also lower in co-culture than mono-culture (p = 0.0197). The only cytosolic flux that increased in co-culture vs. mono-culture was that of ethanol (p = 0.0156), however the magnitude of this difference (0.03 mmol/gDW h) was lesser than the differences between mono- and co-culture of lactate (0.33 mmol/gDW h) and succinate (0.10 mmol/gDW h).

It is possible that ethanol flux increases in co-culture because formate, a putative inhibitor of AGF growth which is produced during production of ethanol *via* the cytosolic pyruvate formate lyase (PFL) pathway, is taken up by methanogens. Production of ethanol regenerates two NAD+ from NADH per pyruvate, whereas production of lactate generates only one, giving the AGF incentive to produce ethanol over lactate if accumulation of inhibitory formate is not an issue. This may also explain why AGF redirect more flux through the hydrogenosome in co-culture with methanogens; more ATP can be generated without accumulating inhibitory formate [21], facilitating faster AGF growth and polysaccharide deconstruction. However, the uncertain and likely degenerate mechanisms of oxidized cofactor regeneration in the hydrogenosome [43] preclude definitive explanation of increased hydrogenosomal fluxes in co-culture.

In co-cultures on glucose, significant differences in metabolite fluxes were the same as those described in Fig. 8, with the exception that succinate fluxes did not differ significantly between mono- and co-culture on glucose (Additional file 9).

#### Quantification of *C. churrovis* external metabolic fluxes highlights gaps in understanding of the AGF hydrogenosome

While a recent description of the AGF hydrogenosome in *N. lanati* showed transcription of several pathways for ATP and hydrogen production [43], analysis of the fluxes of formate, acetate, and ethanol supported the hypothesis initially proposed by Boxma et al. [44] that PFL is the dominant pathway in the hydrogenosome, and flux through other pathways is negligible. Because PFL is present in both the cytosol and the hydrogenosome (Fig. 8**)**, the ratio of formate to ethanol plus acetate produced by the AGF will be unity only if acetate production in the hydrogenosome is always coupled to formate production, implying that only the PFL pathway carries significant flux in the hydrogenosome.

**Fig. 9:**
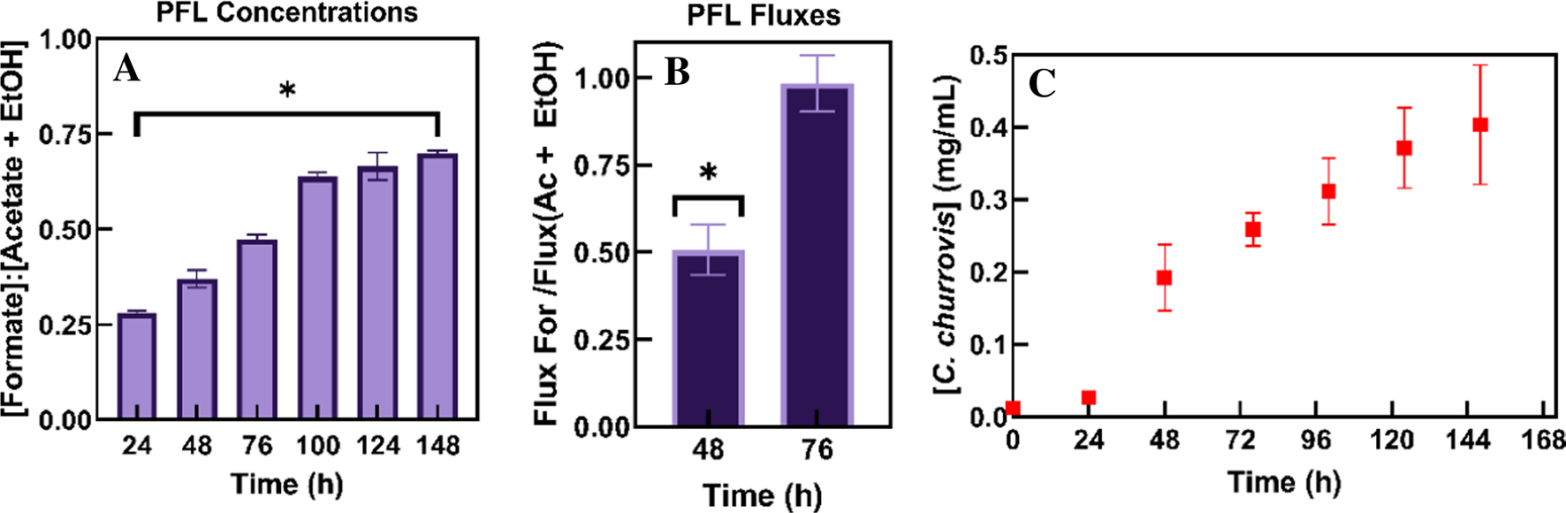
Mono-culture fluxes in MB on glucose suggest that *C. churrovis* hydrogenosome pathways differ from widely accepted PFL model. At all timepoints, the ratio of formate to acetate plus ethanol concentrations produced during growth differs significantly from unity (**A**) (p < 0.0015 for all timepoints). The ratio of formate flux to acetate plus ethanol flux differs significantly from unity during mid-exponential phase growth (48 h, p = 0.0070), but not during late-exponential phase growth (76 h, p = 0.7628) (**B**), suggesting that PFL may dominate hydrogenosome flux only after substrate is depleted and/or inhibitory metabolites have accumulated. Growth curve included (**C**) for reference of growth phases; 76 h and before was considered exponential phase, and all timepoints after were considered stationary phase

This has been observed in at least two AGF to date [43, 44]; however, as shown in Fig. 9B, the ratio of formate to acetate plus ethanol fluxes was significantly different from unity during the phase of constant growth (48 h) for *C. churrovis* (p = 0.0070). Further, the ratio of formate to acetate plus ethanol concentrations was significantly different from unity throughout growth on both substrates (Fig. 9A**)** (p < 0.0015 for all timepoints), suggesting that PFL is not the sole dominant pathway in the hydrogenosome in *C. churrovis*. However, during late-exponential growth (76 h, Fig. 9B**)**, the ratio of formate to acetate plus ethanol flux did not differ significantly from unity (p = 0.7628), suggesting that hydrogenosome flux is dynamic and highly regulated, and PFL may dominate late in *C. churrovis* growth curves. In a separate study, all hydrogenosome components transcribed in *N. lanati* were also detected in *C. churrovis* [45], however, *N. lanati* showed PFL dominance throughout growth while *C. churrovis* did not.

The regulation and relative utilization of other hydrogenosomal pathways present in AGF genomes and transcriptomes, such as pyruvate:ferrodoxin oxidorectucase (PFO) [46], a bifurcating hydogenase, and a putative ATP synthase remains unclear. Previous flux balance analysis predictions suggest that observed hydrogen fluxes are much lower than would be expected if all transcribed hydrogenosomal pathways could carry flux unregulated [43]. To characterize the AGF hydrogenosome to the level required for predictable degradation and conversion of biomass in co-cultures, accurate measurement of steady-state fluxes during chemostat growth may be necessary. Existing pressure-based methods of AGF quantification, even in mono-culture with high temporal resolution [14], are not suitable to quantify AGF concentration and fluxes in a chemostat; however, the method we present here is.

## Conclusions

Microbial communities can be leveraged to perform virtually infinite targeted chemical transformations, and new methods to track their performance in controlled systems are sorely needed. Individual species concentrations are critical metrics that enable many other analyses to understand communities but are challenging to obtain. Here, we have developed a method to obtain these metrics in biotechnologically promising co-cultures of biomass-degrading AGF and methanogens. While previous literature speculates that polysaccharide degradation and AGF growth is accelerated in co-culture with methanogens, methods have not been available to directly test this hypothesis.

With the method we have presented here for rapid quantification of non-rhizoidal AGF and methanogens in co-culture, we have demonstrated significant increases in AGF growth rate and xylan and glucose degradation rate in co-culture with a methanogen. Further, quantitative differences in AGF metabolic fluxes suggest a shift towards more energy-generating hydrogenosome flux in co-culture, however the highlighted uncertainties in the AGF hydrogenosome preclude definitive explanations for this shift. Detailed analyses of AGF that integrate flux measurements with transcriptomics and/or proteomics are likely necessary to characterize the AGF hydrogenosome and unlock their potential for predictable deployment in biotechnology applications; the method presented here is readily extendable to continuous or semi-batch systems for steady-state fluxomics to meet this need.

Importantly, this method may be modified and applied to co-cultures of other organisms with or without autofluorescence for detailed characterization of each organism’s growth, flux, and other metrics that facilitate design and deployment of microbial communities with predictable, tunable functions.

## Supplementary Information

**Additional file 1**: Images (left) and micrographs (right) of rhizoidal AGF *N. lanati* (top) in biofilm-like morphology and non-rhizoidal AGF *C. churrovis* (bottom) in well-mixed cell suspension. Both cultures shown here grown in Medium B on soluble sugars. The *C. churrovis* culture is amenable to growth tracking via optical density of small culture samples, while the *N. lanati* culture is not.

**Additional file 2**: Comparison of *C. churrovis* metabolic EC numbers with the rest of the AGF phylum (Neocallimastigomycota) and with *N. lanati* in particular shows significant similarity in metabolic potential between *C. churrovis* and the rest of the AGF. Bottom row represents the total EC numbers present only in the indicated organism, relative to all of that organism’s EC numbers.

**Additional file 3**: Medium B (MB) formula and protocol. Methanogen mono-cultures receive the peptone and yeast extract additives, while co-cultures and AGF mono-cultures do not.

**Additional file 4**: Mathematical workflow for calculating absorbance and associated uncertainty of each species (A: AGF; B: methanogen) from total co-culture fluorescence (F) and absorbance (Abs) signals. ε is the pure species absorbance per cell and Ƒ is the pure species normalized fluorescence intensity per cell.

**Additional file 5**: The fluorescence intensity of aliquots of Pacific Blue Dye in dimethyl sulfoxide (100 µg/L) stored at -20 ºC did not significantly change over 15 months of storage, indicating its utility as a standard for fluorescence normalization. The slope of the regression of fluorescence intensity vs. time (in weeks) is not significantly different from zero (p = 0.1366), suggesting that fluorescence remains constant over the time period shown. Dotted lines represent the 95% confidence interval of the regression.

**Additional file 6**: The fluorescence intensity of *M. thaueri* cell pellets did not significantly increase when lysed according to the protocol outlined by Peck (Appl Environ Microbiol. 1989; 55:940-945) relative to unlysed (A) (paired t-test p = 0.3229). The fluorescence intensity of *M. thaueri* pellets did not scale linearly with concentration when diluted with concentrated *C. churrovis* (B), suggesting that *C. churrovis* may interfere with the fluorescence of *M. thaueri* pellets, and the combined pellet and supernatant samples of co-cultures should be used to quantify methanogens in co-culture with AGF.

**Additional file 7**: Total culture absorbance (A), *M. thaueri* fluorescence + absorbance (B), *C. churrovis* concentration (C), and glucose consumption (D) curves from co-cultures inoculated with seven day-old *M. thaueri* culture. While methanogen growth occurred (B), neither the growth rate (C) nor glucose consumption rate (D) of C. churrovis increased in co-culture. The fluorescence of the methanogen does not diverge relative to the absorbance (B) because these slow-growing co-cultures did not fully reach stationary phase. All of these results differ from the glucose co-cultures presented in Additional File 8, in which a 48 hour-old *M. thaueri* culture was used for inoculation. Dotted lines represent the 95% confidence interval of each regression. The p-values in panels C and D represents a test for significant difference in the values of the slopes of the two regressions.

**Additional file 8**: Total culture absorbance (A), *M. thaueri* fluorescence + absorbance (B), *C. churrovis* concentration (C) and accumulated pressure (D) curves show that both the growth rate of *C. churrovis* and the rate of gas production are significantly increased in co-cultures with *M. thaueri* grown on glucose, relative to monocultures. Panel B shows the divergence of *M. thaueri* fluorescence relative to absorbance in stationary phase also observed in mono-culture; the absorbance of the methanogen was assumed to remain constant after the absorbance of the co-culture stops increasing (96h and after). Dotted lines represent the 95% confidence interval of each regression. The p-values in panels C and D represents a test for significant difference in the values of the slopes of the two regressions.

**Additional file 9**: Metabolite profiles (A) and cell mass-normalized fluxes (B) reveal significant upregulation (*U) of acetate and ethanol fluxes, and significant downregulation (*D) of lactate flux in co-cultures. Fumarate is an intermediate to succinate production, and it is consumed more quickly in co-cultures. Formate and hydrogen are consumed by M. thaueri and therefore do not accumulate in co-cultures. While glucose is consumed more quickly in co-culture, the flux of glucose into *C. churrovis* is equal in mono- and co-cultures. Dotted lines represent the 95% confidence interval of each regression. The p-value in panel (i) represents a test for significant difference in the values of the slopes of the two regressions.

## Data Availability

All data generated or analyzed during this study are included in this published article and its supplementary information files.

## Footnotes

1 Formate utilization was observed in *M. thaueri* mono-cultures (not shown) and co-cultures, despite a previous contrary description [28]. Formate dehydrogenase subunits are present in the only published *M. thaueri* genome on NCBI.
