# Supplementary material for: Non-destructive quantification of anaerobic gut fungi and methanogens in co-culture reveals increased fungal growth rate and changes in metabolic flux relative to mono-culture
Source: Microb Cell Fact. 2021 Oct 18;20:199. doi: 10.1186/s12934-021-01684-2 (PMC8522008; doi:10.1186/s12934-021-01684-2)
Supplement: Supplementary file 1 — Additional file 1: Images (left) and micrographs (right) of rhizoidal AGF N. lanati (top) in biofilm-like morphology and non-rhizoidal AGF C. churrovis (bottom) in well-mixed cell suspension. Both cultures shown here grown in Medium B on soluble sugars. The C. churrovis culture is amenable to growth tracking via optical density of small culture samples, while the N. lanati culture is not. [file 12934_2021_1684_MOESM1_ESM.docx]

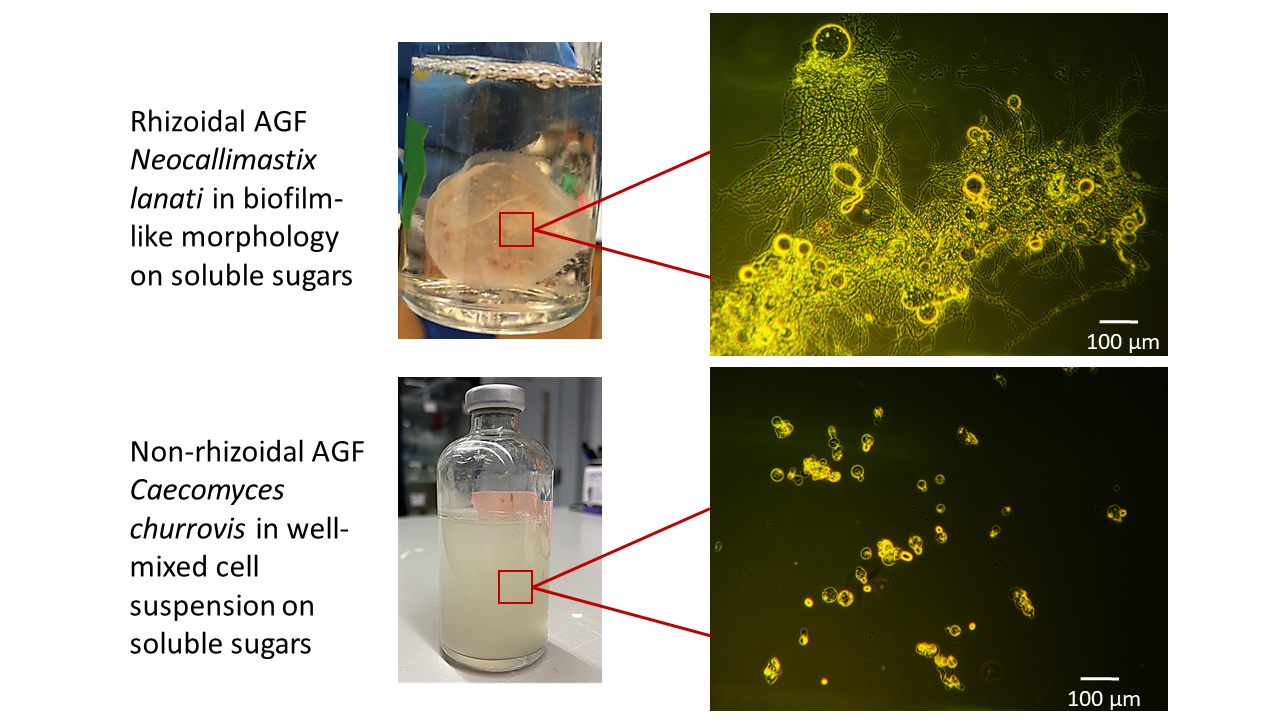


`

**Additional File 1)** Images (left) and micrographs (right) of rhizoidal AGF *N. lanati* (top) in biofilm-like morphology and non-rhizoidal AGF *C. churrovis* (bottom) in well-mixed cell suspension. Both cultures shown here grown in Medium B on soluble sugars. The *C. churrovis* culture is amenable to growth tracking via optical density of small culture samples, while the *N. lanati* culture is not.
