# Supplementary material for: Non-destructive quantification of anaerobic gut fungi and methanogens in co-culture reveals increased fungal growth rate and changes in metabolic flux relative to mono-culture
Source: Microb Cell Fact. 2021 Oct 18;20:199. doi: 10.1186/s12934-021-01684-2 (PMC8522008; doi:10.1186/s12934-021-01684-2)
Supplement: Supplementary file 2 — Additional file 2: Comparison of C. churrovis metabolic EC numbers with the rest of the AGF phylum (Neocallimastigomycota) and with N. lanati in particular shows significant similarity in metabolic potential between C. churrovis and the rest of the AGF. Bottom row represents the total EC numbers present only in the indicated organism, relative to all of that organism’s EC numbers. [file 12934_2021_1684_MOESM2_ESM.docx]

**Additional File 2**) Comparison of *C. churrovis* metabolic EC numbers with the rest of the AGF phylum (Neocallimastigomycota) and with *N. lanati* in particular shows significant similarity in metabolic potential between *C. churrovis* and the rest of the AGF. Bottom row represents the total EC numbers present only in the indicated organism, relative to all of that organism’s EC numbers.

|  | *C. churrovis* only (no other AGF) | *C. churrovis* but not *N. lanati* | *N. lanati* but not  *C. churrovis* |
| --- | --- | --- | --- |
| Amino acids | 1 | 5 | 6 |
| Carbon | 2 | 3 | 12 |
| Glycans | 2 | 1 | 0 |
| Lipids | 0 | 4 | 6 |
| Nucleotides | 1 | 2 | 4 |
| Secondary met. | 0 | 0 | 1 |
| Terpenoids | 2 | 2 | 0 |
| Vitamins | 3 | 3 | 3 |
| Uncertain | 11 | 33 | 41 |
| Total | 22 | 53 | 73 |
| **Percent of total organism ECs** | **3.1%** | **7.6%** | **10.1%** |
