# Supplementary material for: Non-destructive quantification of anaerobic gut fungi and methanogens in co-culture reveals increased fungal growth rate and changes in metabolic flux relative to mono-culture
Source: Microb Cell Fact. 2021 Oct 18;20:199. doi: 10.1186/s12934-021-01684-2 (PMC8522008; doi:10.1186/s12934-021-01684-2)
Supplement: Supplementary file 3 — Additional file 3: Medium B (MB) formula and protocol. Methanogen mono-cultures receive the peptone and yeast extract additives, while co-cultures and AGF mono-cultures do not. [file 12934_2021_1684_MOESM3_ESM.pdf]

Defined Medium B

PA Leggieri April 19, 2021

Adapted from the protocol developed by Marcus Benyamin at ARL

MB Base Solution (to be autoclaved), 2-3 day prep

1. In a 2.8 L boiling flask, add 1.2 L of Milli-Q water (to account for evaporation).
2. Boil for 15-20 minutes.
3. While flushing with CO_2, measure 800 mL of the freshly boiled water and transfer to a 1 L bottle (there should be some left behind).
4. While bubbling the bottle with CO_2, add 100 mL MB Salts solution and 1 mL resazurin
5. Bubble for ~30 minutes.
6. Add the 50 mL Na_2CO_3 + cysteine-HCl solution. The solution will turn opaque blue.
7. Continue to bubble with CO_2 until the solution is translucent hot pink (~40 minutes).
8. At this point, the media can be tightly capped, and transferred to the anaerobic chamber to continue reducing overnight. **Loosen the cap once the bottle is in the chamber.**
9. While moving the media through the passbox, move all tubes/bottles into the chamber to become anoxic **overnight, as well as an additional empty 80 mL serum bottle**.
10. Aliquot the media and seal the tubes/bottles in the chamber (9 mL into each tube and 38 mL into each bottle). Seal the empty 80mL serum bottle as well. Remove and autoclave.
11. After autoclaving, the carbonate will have crashed out of solution. Shake vigorously and place in the refrigerator for a few hours or overnight to dissolve.

MB Additives Solution (to be sterile filtered and added directly to culture vessels)

1. To a beaker, add the following additives:
    a. 10 mL coenzyme M solution
    b. 10 mL Gibco pen/strep solution (optional)
    c. 10 mL MOPS buffer solution
    d. 10 mL ATCC trace mineral solution
    e. 10 mL ATCC trace vitamin solution
    f. 5 mL hemin solution
    g. **Optional and not recommended for HPLC use**: 10 mL fatty acid solution
2. Sterile filter the additives solution directly into the empty autoclaved, sealed, anoxic serum bottle using a pink needle. If the filter is too clogged with hemin to continue filtering, replace it with a new one and continue filtering.

Combining Salts and Additives Solutions at Point of Culture

Before inoculation, add 0.55 mL of additives to the 9 mL of MB Base solution in each tube. Scale this ratio appropriately for larger culture volumes.

Medium B Components

PA Leggieri April 19, 2021

- MB Salts solution (10x)
  - 6.8 g/L KH_2PO_4
  - 6.0 g/L KCl
  - 6.0 g/L NaCl
  - 5.0 g/L $MgSO_4$ * $7H_2O$
  - 2.0 g/L $CaCl_2$ * $2H_2O$
  - 5.4 g/L NH_4Cl

*These concentrations are in each individual solution, not the final combined MB media.

- Coenzyme M solution (4 g/L)
- MOPS buffer solution {3-(*N*-morpholino)propanesulfonic acid} (100 g/L)
- Resazurin solution (1 g/L)
- Hemin solution (0.5 g/L with 10 mL/L ethanol and 2 g/L NaOH)
- Na_2CO_3 (80 g/L) + cysteine-HCl (20 g/L)
- Fatty acid solution (optional, not recommended for HPLC use)
  - Acetic acid (6.85 mL/L)
  - Propionic acid (3.0 mL/L)
  - Butyric acid (1.84 mL/L)
  - Methylbutyric acid (0.55 mL/L)
  - Isobutyric acid (0.47 mL/L)
  - Valeric acid (0.55 mL/L)
  - Isovaleric acid (0.55 mL/L)
  - NaOH (g g/L)

- ATCC trace mineral solution (can make in house)
  - EDTA (0.5 g/L)
  - $MgSO_4$ * $7H_2O$ (3.0 g/L)
  - $MnSO_4$ * H_2O (0.5 g/L)
  - NaCl (1.0 g/L)
  - $FeSO_4$ * $7H_2O$ (0.1 g/L)
  - $Co(NO_3)_2$ * $6H_2O$ (0.1 g/L)
  - $CaCl_2$ (anhydrous) (0.1 g/L)
  - $ZnSO_4$ * $7H_2O$ (0.1 g/L)
  - $CuSO_4$ * $5H_2O$ (0.010 g/L)
  - $AlK(SO_4)_2$ (anhydrous) (0.010 g/L)
  - H_3BO_3 (0.010 g/L)
  - Na_2MoO_4 * $2H_2O$ (0.010 g/L)
  - Na_2SeO_3 (anhydrous) (0.001 g/L)
  - Na_2WO_4 * $2H_2O$ (0.010 g/L)
  - $NiCl_2$ * $6H_2O$ (0.020 g/L)

- ATCC trace vitamin solution (can make in house)
  - Folic acid (2.0 mg/L)
  - Pyridoxine hydrochloride (10.0 mg/L)
  - Riboflavin (5.0 mg/L)
  - Biotin (2.0 mg/L)
  - Thiamine (5.0 mg/L)
  - Nicotinic acid (5.0 mg/L)
  - Calcium Pantothenate (5.0 mg/L)
  - Vitamin B12 (0.1 mg/L)
  - p-Aminobenzoic acid (5.0 mg/L)
  - Thioctic acid (5.0 mg/L)
  - Monopotassium phosphate (900.0 mg/L)

- The following two components may be added to the MB Additives if a fully defined formulation is not necessary (e.g., to support growth of some methanogens):
  - 10 mL peptone solution (100 g/L peptone, tryptone, or bacto-casitone)
  - 10 mL yeast extract solution (50 g/L)

Additional File 3) Medium B (MB) formula and protocol. Methanogen mono-cultures receive the peptone and yeast extract additives, while co-cultures and AGF mono-cultures do not.
