# Supplementary material for: Non-destructive quantification of anaerobic gut fungi and methanogens in co-culture reveals increased fungal growth rate and changes in metabolic flux relative to mono-culture
Source: Microb Cell Fact. 2021 Oct 18;20:199. doi: 10.1186/s12934-021-01684-2 (PMC8522008; doi:10.1186/s12934-021-01684-2)

**Additional File 4)** Mathematical workflow for calculating absorbance and associated uncertainty of each species (A: AGF; B: methanogen) from total co-culture fluorescence (F) and absorbance (Abs) signals. ε is the pure species absorbance per cell and $\mathcal{F}$is the pure species normalized fluorescence intensity per cell.

Fluorescence


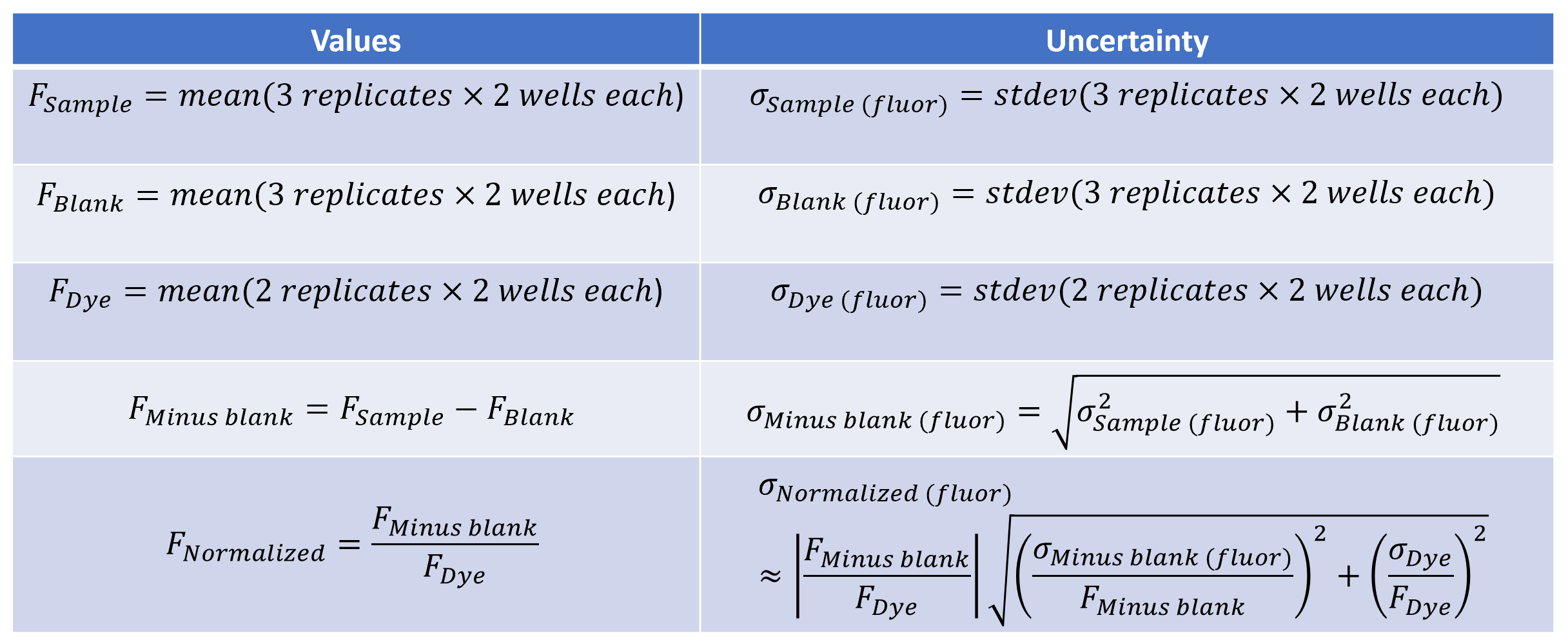


Absorbance
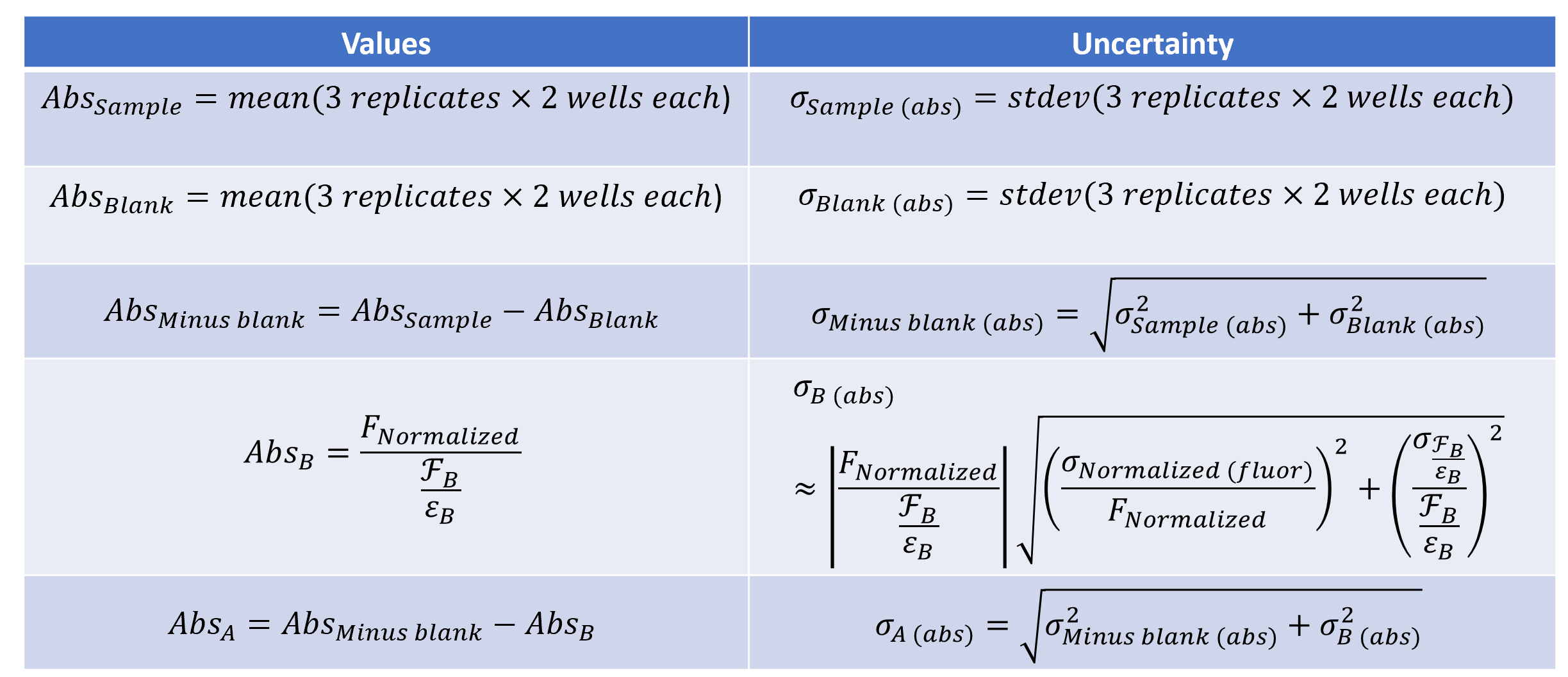

Supplement: Supplementary file 4 — Additional file 4: Mathematical workflow for calculating absorbance and associated uncertainty of each species (A: AGF; B: methanogen) from total co-culture fluorescence (F) and absorbance (Abs) signals. ε is the pure species absorbance per cell and Ƒ is the pure species normalized fluorescence intensity per cell. [file 12934_2021_1684_MOESM4_ESM.docx]
