# Supplementary material for: Non-destructive quantification of anaerobic gut fungi and methanogens in co-culture reveals increased fungal growth rate and changes in metabolic flux relative to mono-culture
Source: Microb Cell Fact. 2021 Oct 18;20:199. doi: 10.1186/s12934-021-01684-2 (PMC8522008; doi:10.1186/s12934-021-01684-2)
Supplement: Supplementary file 5 — Additional file 5: The fluorescence intensity of aliquots of Pacific Blue Dye in dimethyl sulfoxide (100 µg/L) stored at -20 ºC did not significantly change over 15 months of storage, indicating its utility as a standard for fluorescence normalization. The slope of the regression of fluorescence intensity vs. time (in weeks) is not significantly different from zero (p = 0.1366), suggesting that fluorescence remains constant over the time period shown. Dotted lines represent the 95% confidence interval of the regression. [file 12934_2021_1684_MOESM5_ESM.docx]

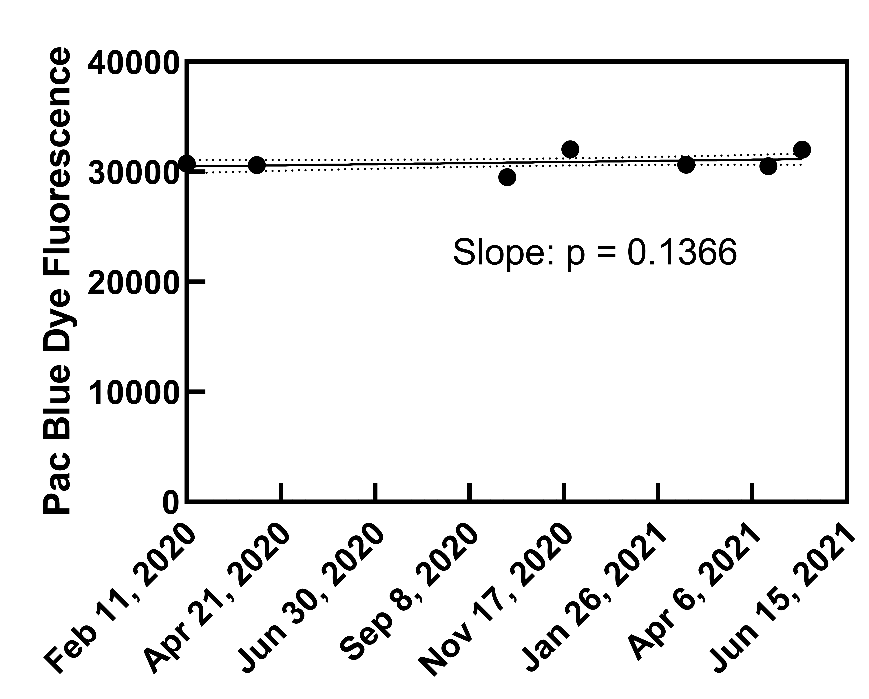


**Additional File 5.** The fluorescence intensity of aliquots of Pacific Blue Dye in dimethyl sulfoxide (100 µg/L) stored at -20 ºC did not significantly change over 15 months of storage, indicating its utility as a standard for fluorescence normalization. The slope of the regression of fluorescence intensity vs. time (in weeks) is not significantly different from zero (p = 0.1366), suggesting that fluorescence remains constant over the time period shown. Dotted lines represent the 95% confidence interval of the regression.
