# Supplementary material for: Non-destructive quantification of anaerobic gut fungi and methanogens in co-culture reveals increased fungal growth rate and changes in metabolic flux relative to mono-culture
Source: Microb Cell Fact. 2021 Oct 18;20:199. doi: 10.1186/s12934-021-01684-2 (PMC8522008; doi:10.1186/s12934-021-01684-2)

**Additional File 6.** The fluorescence intensity of *M. thaueri* cell pellets did not significantly increase when lysed according to the protocol outlined by Peck *(Appl Environ Microbiol*. 1989; 55:940-945*)* relative to unlysed (A) (paired t-test p = 0.3229). The fluorescence intensity of *M. thaueri* pellets did not scale linearly with concentration when diluted with concentrated *C. churrovis* (B), suggesting that *C. churrovis* may interfere with the fluorescence of *M. thaueri* pellets, and the combined pellet and supernatant samples of co-cultures should be used to quantify methanogens in co-culture with AGF.


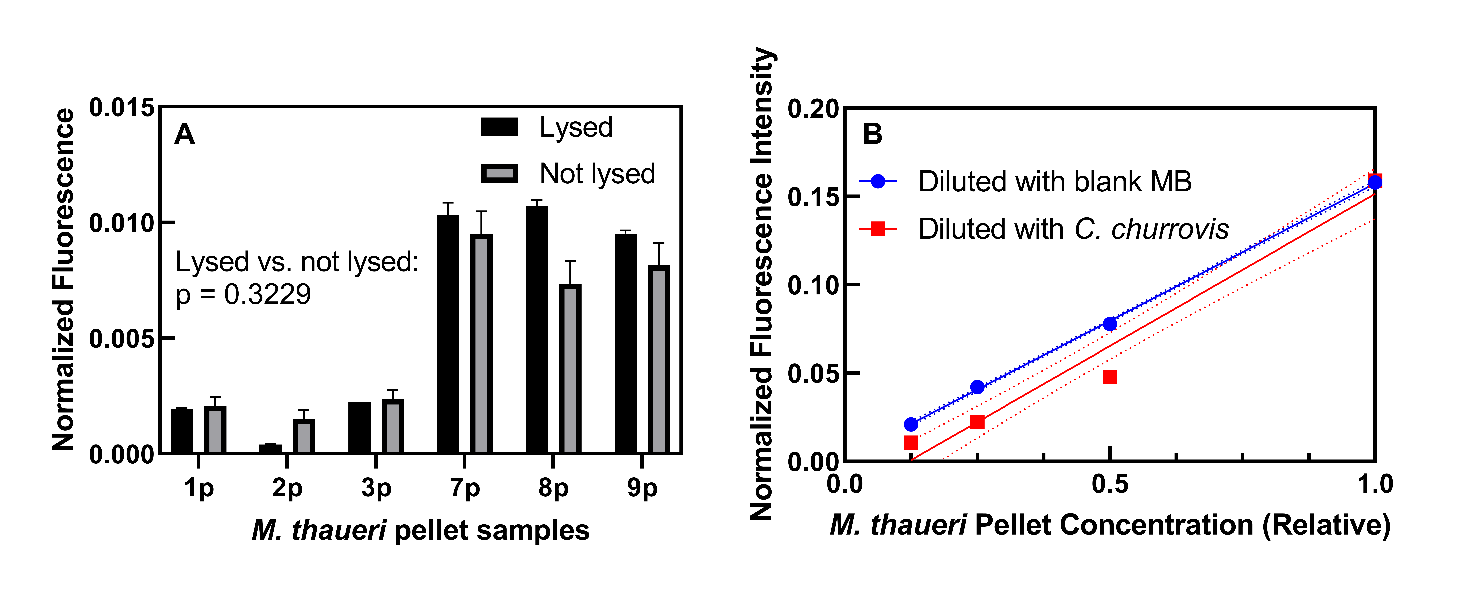

Supplement: Supplementary file 6 — Additional file 6: The fluorescence intensity of M. thaueri cell pellets did not significantly increase when lysed according to the protocol outlined by Peck (Appl Environ Microbiol. 1989; 55:940-945) relative to unlysed (A) (paired t-test p = 0.3229). The fluorescence intensity of M. thaueri pellets did not scale linearly with concentration when diluted with concentrated C. churrovis (B), suggesting that C. churrovis may interfere with the fluorescence of M. thaueri pellets, and the combined pellet and supernatant samples of co-cultures should be used to quantify methanogens in co-culture with AGF. [file 12934_2021_1684_MOESM6_ESM.docx]
