# Supplementary material for: Non-destructive quantification of anaerobic gut fungi and methanogens in co-culture reveals increased fungal growth rate and changes in metabolic flux relative to mono-culture
Source: Microb Cell Fact. 2021 Oct 18;20:199. doi: 10.1186/s12934-021-01684-2 (PMC8522008; doi:10.1186/s12934-021-01684-2)

**Additional File 7)** Total culture absorbance (A), *M. thaueri* fluorescence + absorbance (B), *C. churrovis* concentration (C), and glucose consumption (D) curves from co-cultures inoculated with seven day-old *M. thaueri* culture. While methanogen growth occurred (B), neither the growth rate (C) nor glucose consumption rate (D) of *C. churrovis* increased in co-culture. The fluorescence of the methanogen does not diverge relative to the absorbance (B) because these slow-growing co-cultures did not fully reach stationary phase. All of these results differ from the glucose co-cultures presented in Additional File 8, in which a 48 hour-old *M. thaueri* culture was used for inoculation. Dotted lines represent the 95% confidence interval of each regression. The p-values in panels C and D represents a test for significant difference in the values of the slopes of the two regressions.


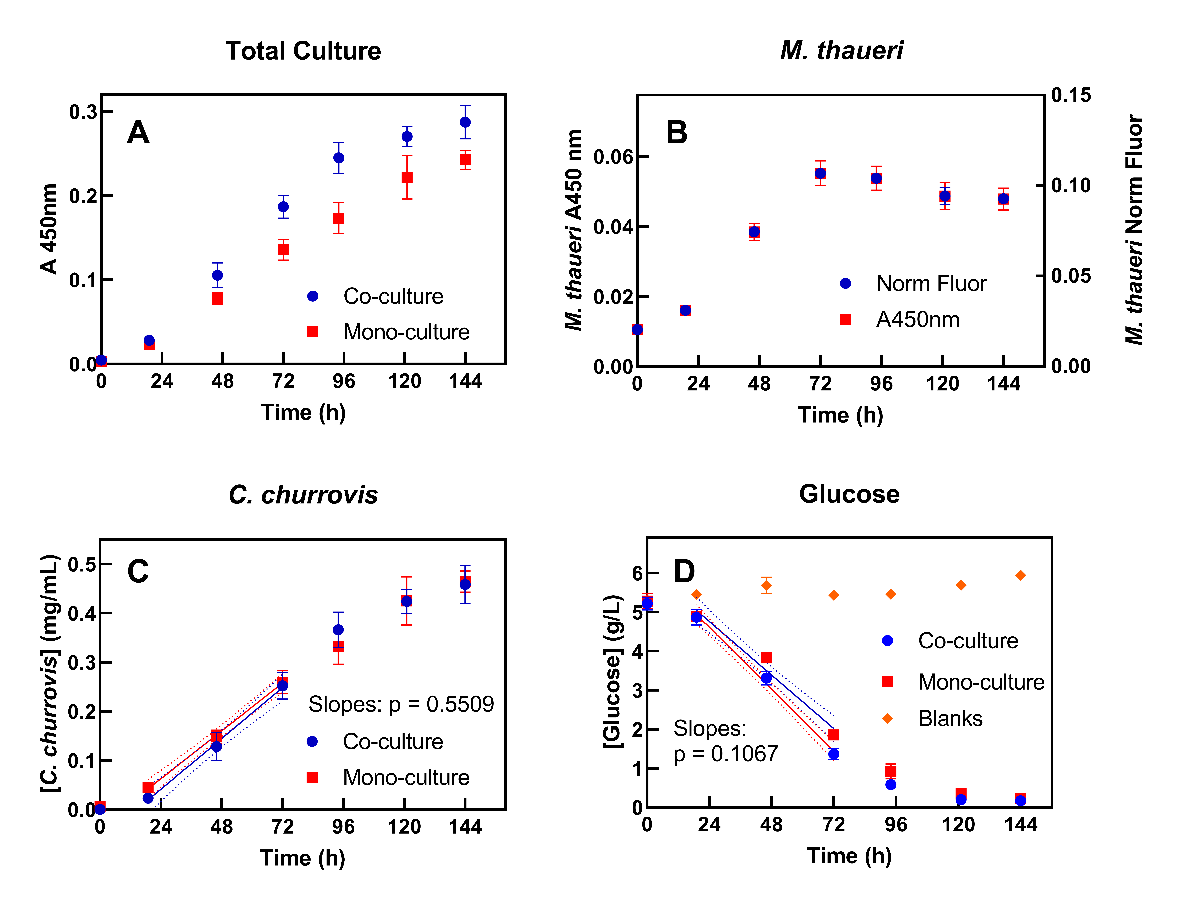

Supplement: Supplementary file 7 — Additional file 7: Total culture absorbance (A), M. thaueri fluorescence + absorbance (B), C. churrovis concentration (C), and glucose consumption (D) curves from co-cultures inoculated with seven day-old M. thaueri culture. While methanogen growth occurred (B), neither the growth rate (C) nor glucose consumption rate (D) of C. churrovis increased in co-culture. The fluorescence of the methanogen does not diverge relative to the absorbance (B) because these slow-growing co-cultures did not fully reach stationary phase. All of these results differ from the glucose co-cultures presented in Additional File 8, in which a 48 hour-old M. thaueri culture was used for inoculation. Dotted lines represent the 95% confidence interval of each regression. The p-values in panels C and D represents a test for significant difference in the values of the slopes of the two regressions. [file 12934_2021_1684_MOESM7_ESM.docx]
