# Supplementary material for: Non-destructive quantification of anaerobic gut fungi and methanogens in co-culture reveals increased fungal growth rate and changes in metabolic flux relative to mono-culture
Source: Microb Cell Fact. 2021 Oct 18;20:199. doi: 10.1186/s12934-021-01684-2 (PMC8522008; doi:10.1186/s12934-021-01684-2)
Supplement: Supplementary file 8 — Additional file 8: Total culture absorbance (A), M. thaueri fluorescence + absorbance (B), C. churrovis concentration (C) and accumulated pressure (D) curves show that both the growth rate of C. churrovis and the rate of gas production are significantly increased in co-cultures with M. thaueri grown on glucose, relative to monocultures. Panel B shows the divergence of M. thaueri fluorescence relative to absorbance in stationary phase also observed in mono-culture; the absorbance of the methanogen was assumed to remain constant after the absorbance of the co-culture stops increasing (96h and after). Dotted lines represent the 95% confidence interval of each regression. The p-values in panels C and D represents a test for significant difference in the values of the slopes of the two regressions. [file 12934_2021_1684_MOESM8_ESM.docx]

**Additional File 8)** Total culture absorbance (A), *M. thaueri* fluorescence + absorbance (B), *C. churrovis* concentration (C) and accumulated pressure (D) curves show that both the growth rate of *C. churrovis* and the rate of gas production are significantly increased in co-cultures with *M. thaueri* grown on glucose, relative to monocultures. Panel B shows the divergence of *M. thaueri* fluorescence relative to absorbance in stationary phase also observed in mono-culture; the absorbance of the methanogen was assumed to remain constant after the absorbance of the co-culture stops increasing (96h and after). Dotted lines represent the 95% confidence interval of each regression. The p-values in panels C and D represents a test for significant difference in the values of the slopes of the two regressions.

*
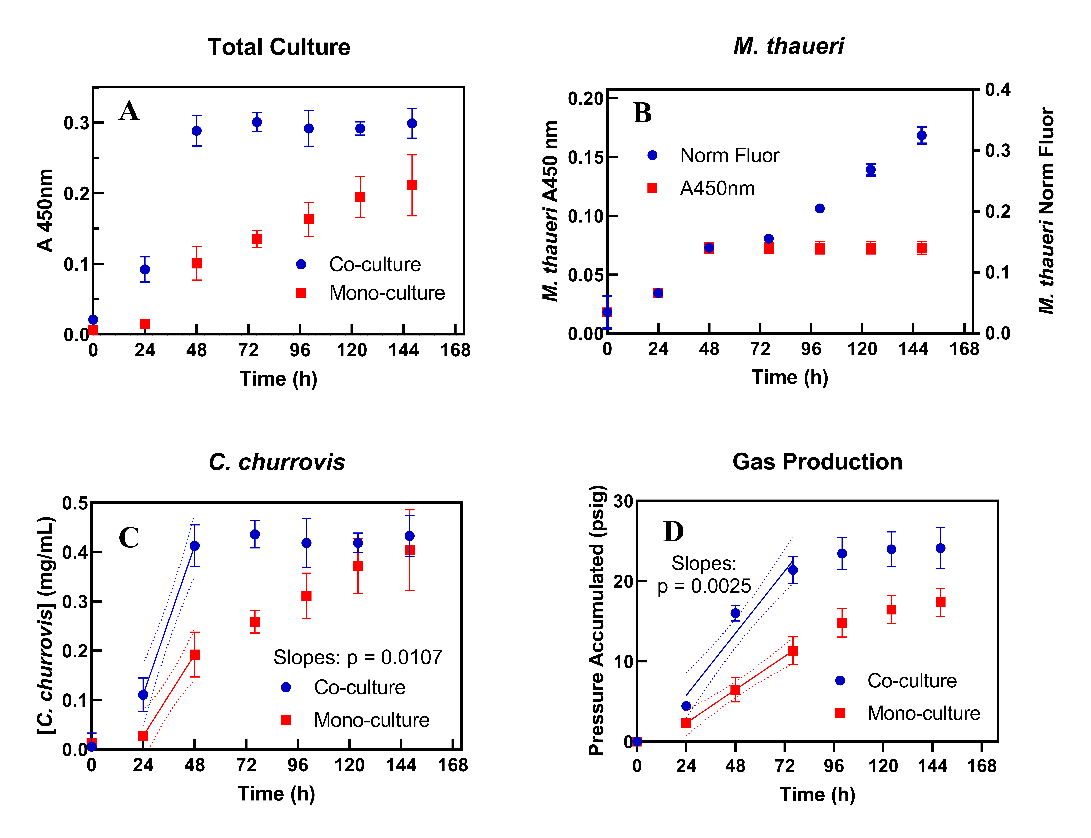
*
