# Supplementary material for: Non-destructive quantification of anaerobic gut fungi and methanogens in co-culture reveals increased fungal growth rate and changes in metabolic flux relative to mono-culture
Source: Microb Cell Fact. 2021 Oct 18;20:199. doi: 10.1186/s12934-021-01684-2 (PMC8522008; doi:10.1186/s12934-021-01684-2)

**Additional File 9)** Metabolite profiles (A) and cell mass-normalized fluxes (B) reveal significant upregulation (*U) of acetate and ethanol fluxes, and significant downregulation (*D) of lactate flux in co-cultures. Fumarate is an intermediate to succinate production, and it is consumed more quickly in co-cultures. Formate and hydrogen are consumed by *M. thaueri* and therefore do not accumulate in co-cultures. While glucose is consumed more quickly in co-culture, the flux of glucose into *C. churrovis* is equal in mono- and co-cultures. Dotted lines represent the 95% confidence interval of each regression. The p-value in panel (i) represents a test for significant difference in the values of the slopes of the two regressions.


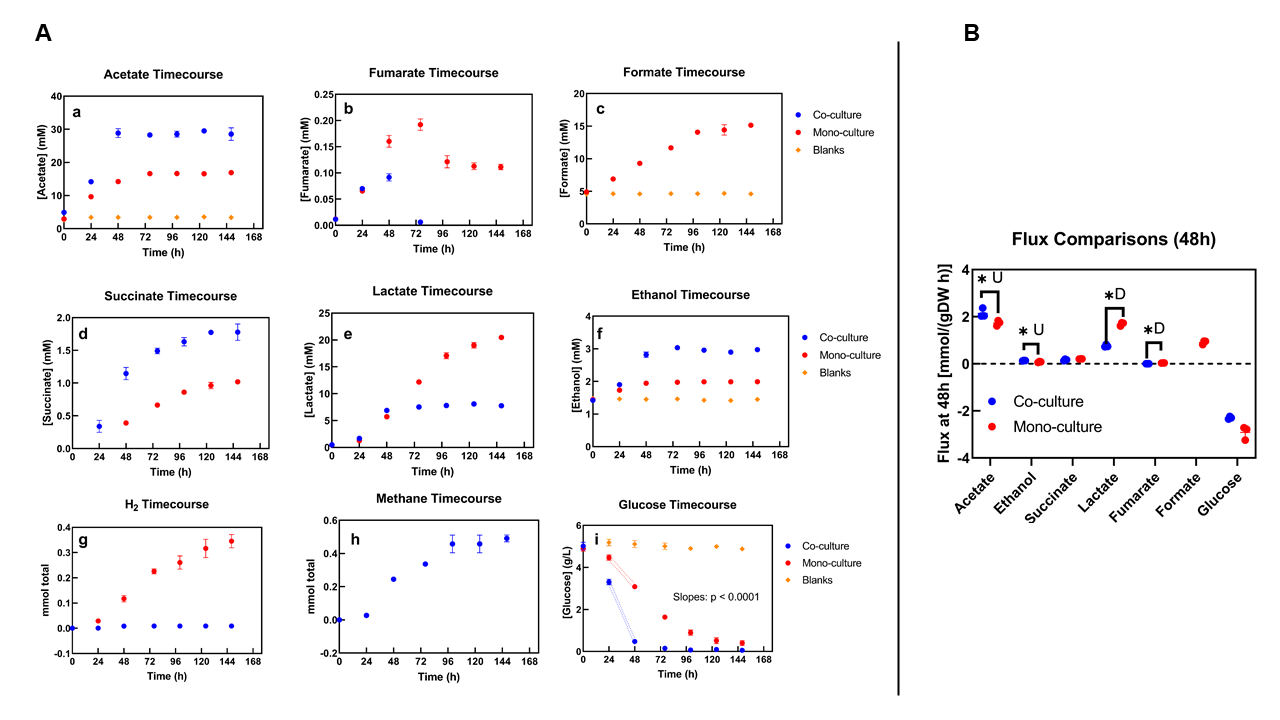

Supplement: Supplementary file 9 — Additional file 9: Metabolite profiles (A) and cell mass-normalized fluxes (B) reveal significant upregulation (*U) of acetate and ethanol fluxes, and significant downregulation (*D) of lactate flux in co-cultures. Fumarate is an intermediate to succinate production, and it is consumed more quickly in co-cultures. Formate and hydrogen are consumed by M. thaueri and therefore do not accumulate in co-cultures. While glucose is consumed more quickly in co-culture, the flux of glucose into C. churrovis is equal in mono- and co-cultures. Dotted lines represent the 95% confidence interval of each regression. The p-value in panel (i) represents a test for significant difference in the values of the slopes of the two regressions. [file 12934_2021_1684_MOESM9_ESM.docx]
